# 3-(1,2,3-Triazol-4-yl)-β-Carbolines and 3-(1*H*-Tetrazol-5-yl)-β-Carbolines: Synthesis and Evaluation as Anticancer Agents

**DOI:** 10.3390/ph15121510

**Published:** 2022-12-03

**Authors:** João L. P. Ribeiro, Joana B. Loureiro, Susana M. M. Lopes, Lucília Saraiva, Teresa M. V. D. Pinho e Melo

**Affiliations:** 1 University of Coimbra, Coimbra Chemistry Centre-Institute of Molecular Sciences, Department of Chemistry, 3004-535 Coimbra, Portugal; 2 LAQV/REQUIMTE, Laboratory of Microbiology, Department of Biological Sciences, Faculty of Pharmacy, University of Porto, Rua Jorge Viterbo Ferreira, 228, 4050-313 Porto, Portugal

**Keywords:** β-carbolines, anticancer agents, triazoles, tetrazoles, nitrosoalkenes, hetero-Diels–Alder reaction, tryptamine analogs

## Abstract

Herein, the synthesis and anticancer activity evaluation of a series of novel β-carbolines is reported. The reactivity of nitrosoalkenes towards indole was explored for the synthesis of novel tryptophan analogs where the carboxylic acid was replaced by a triazole moiety. This tryptamine was used in the synthesis of 3-(1,2,3-triazol-4-yl)-β-carbolines via Pictet–Spengler condensation followed by an oxidative step. A library of compounds, including the novel 3-(1,2,3-triazol-4-yl)-β-carbolines as well as methyl β-carboline-3-carboxylate and 3-tetrazolyl-β-carboline derivatives, was evaluated for their antiproliferative activity against colorectal cancer cell lines. The 3-(1*H*-tetrazol-5-yl)-β-carbolines stood out as the most active compounds, with values of half-maximal inhibitory concentration (IC_50_) ranging from 3.3 µM to 9.6 µM against colorectal adenocarcinoma HCT116 and HT29 cell lines. The results also revealed a mechanism of action independent of the p53 pathway. Further studies with the 3-tetrazolyl-β-carboline derivative, which showed high selectivity for cancer cells, revealed IC_50_ values below 8 μM against pancreatic adenocarcinoma PANC-1, melanoma A375, hepatocarcinoma HEPG2, and breast adenocarcinoma MCF-7 cell lines. Collectively, this work discloses the 3-tetrazolyl-β-carboline derivative as a promising anticancer agent worthy of being further explored in future works.

## 1. Introduction

Cancer is one of the leading causes of death worldwide. In 2020, the number of new cases of cancer was estimated to be around 19.3 million, with approximately 10 million deaths globally being attributed to cancer [1]. Moreover, colorectal cancer (CRC) is the third most common cancer type, and one of the deadliest (in 2020, CRC accounted for over 2 million new cancer cases and 1 million deaths worldwide). Therefore, the continuous effort towards the discovery of novel and effective anticancer agents against CRC is of great importance [1].

β-Carbolines are a class of indole-based natural and synthetic compounds bearing a 9*H*-pyrido[3,4-*b*]indole scaffold, with a wide range of biological activities, such as anti-HIV [2,3,4], antibacterial [3,4,5], antimalarial [6,7], and anticancer [8,9], among others [10,11,12]. It is noteworthy to mention their anticancer activity, which has been extensively studied in recent years by several groups, with promising results [5,8,9,13,14,15,16,17,18].

**Scheme 1:**
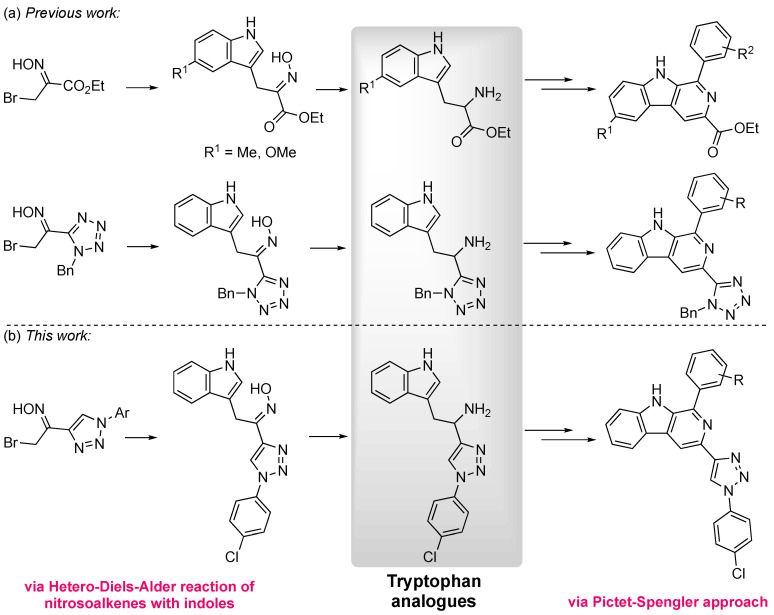
(**a**) Previous work on the synthesis of novel β-carbolines by our group [8]. (**b**) This work: synthesis of 3-triazolyl-β-carbolines.

In recent years, several synthetic methodologies have been applied to the development of novel β-carboline derivatives. However, the most commonly explored synthetic route involves the Pictet–Spengler reaction between an aldehyde and a tryptamine derivative, followed by oxidation of the initially formed tetrahydro-β-carboline [7,19,20]. In our group, the reactivity of nitrosoalkenes towards heterocycles has been extensively studied, and it was thought that it could be explored for the synthesis of functionalized tryptophan analogs [21,22,23]. The nitrosoalkenes are generated in situ through the treatment of α-bromooximes with sodium carbonate, followed by the hetero-Diels–Alder reaction with indoles, subsequent 1,2-oxazine ring-opening, and concomitant rearomatization to afford 3-alkylated indoles incorporating an oxime moiety [24,25]. This open-chain oxime is then reduced to afford the desired tryptamine analog. In fact, the synthesis of a tryptophan analog, where the carboxylic acid was replaced by the bioisosteric tetrazole group, was recently reported by our group, and was used as a building block for the synthesis of novel 3-tetrazolyl-β-carbolines (Scheme 1a) [8]. Using a similar synthetic strategy, 6-substituted-β-carboline-3-carboxylates were also synthesized via the Pictet–Spengler approach, using tryptophan ethyl esters functionalized in the indole moiety (Scheme 1a) [8].

**Figure 1:**
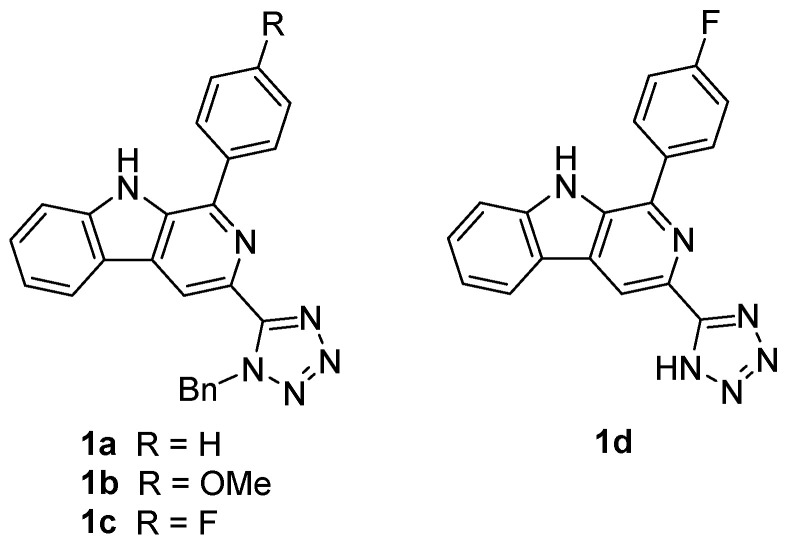
3-Tetrazolyl-β-carbolines obtained from the tetrazolyl tryptophan analog via Pictet–Spengler, followed by oxidation approach.

The activity of the synthesized 3-(1-benzyl-tetrazolyl)-β-carbolines **1a–c** was evaluated against several human cancer cell lines, which evidenced their potential as anticancer agents (Figure 1) [8]. It is noteworthy to mention that the β-carboline derivative **1c**, bearing a 4-fluorophenyl at C-1, was active against ovarian carcinoma (OVCAR-3), leukemia (K-562), renal adenocarcinoma (786-0), and breast adenocarcinoma (MCF-7), with half-maximal inhibitory concentration (IC_50_) values below 1 μM. The corresponding β-carboline **1d** with the unprotected tetrazole ring showed significantly lower activity against most of the cancer cell lines studied, but was, nevertheless, selective and very active against ovarian carcinoma (OVCAR-3, IC_50_ = 0.27 μM). 3-Tetrazolyl-β-carboline **1b**, bearing a 4-methoxyphenyl group at C-1, was also very active against several cancer cell lines, namely breast adenocarcinoma, lung carcinoma (NCI-H460), and ovarian carcinoma, with IC_50_ values between 1.32 and 1.62 µM. In contrast, compound **1a**, containing a phenyl group at C-1, showed poor activity, except against breast cancer cell line (MCF-7), presenting an IC_50_ value of 4.40 µM.

**Figure 2:**
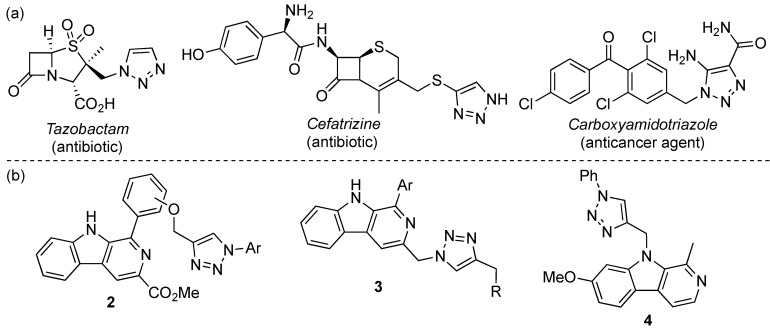
Known drugs (**a**) and β-carbolines with anticancer activity (**b**) containing a 1,2,3-triazole motif.

Over the past decade, 1,2,3-triazoles derivatives have been extensively studied in medicinal chemistry due to their wide range of biological activities [26,27,28,29,30,31], being found in several well-known drugs (Figure 1a) [32,33,34]. In addition to their antifungal [35], antibacterial [36,37,38], anti-HIV [39], and antimalarial [40] activity, these five-membered heterocycles have been widely recognized for their anticancer activity [41,42,43,44]. The great biological potential of 1,2,3-triazoles is related to their high chemical stability, structural rigidity, ability to form hydrogen bonds in the biological environment, strong dipole moment, and the ability to mimic other groups such as amides, esters, and carboxylic acids [45,46]. Recently, reports on the synthesis and biological activity of β-carboline and 1,2,3-triazole hybrids have been disclosed. For instance, compounds with the general structure **2** showed antibacterial activity and high cytotoxicity against the Hela and HepG2 cell lines [47]. On the other hand, β-carbolines **3** [48] and the harmine derivative **4** [49] showed interesting anticancer activity against the MCF-7 cell line (Figure 2b).

Taking our previous promising results into account, we decided to carry out further studies on the anticancer activity of 3-tetrazolyl-β-carbolines, namely against several colon cancer cell lines. Moreover, the first synthesis of a novel tryptophan analog where the carboxylic acid was replaced by a 1,2,3-triazole moiety, as well the synthesis of a library of novel 3-triazolyl-β-carbolines, were carried out (Scheme 1b). The evaluation of the latter β-carbolines, as well as of a range of methyl β-carboline-3-carboxylates, as anticancer agents against human colon cancer cells is also reported.

## 2. Results and Discussion

### 2.1. Chemistry

**Scheme 2:**
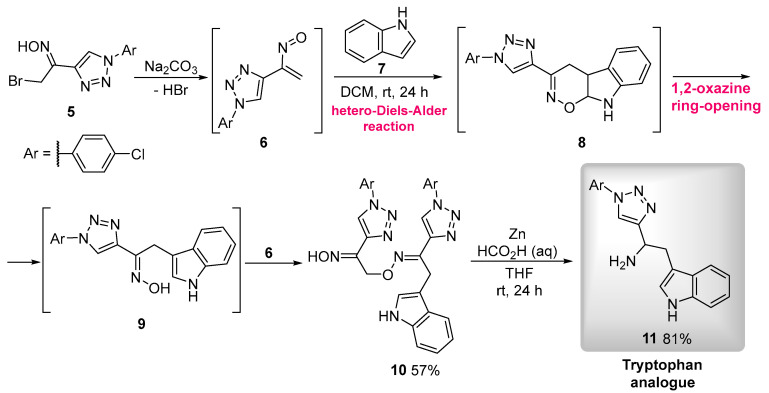
Synthesis of a tryptamine bearing a 1,2,3-triazole group.

In order to develop the proposed 3-triazolyl-β-carboline derivatives, firstly, the α-bromooxime **5** was prepared following previously reported procedures [21]. Treatment of oxime **5** with sodium carbonate led to the in situ formation of the nitrosoalkene **6**, which reacted with indole (**7**) to give 3-alkylated indole **9** via hetero-Diels–Alder reaction, followed by a 1,2-oxazine ring-opening reaction. Oxime **9** reacted further with another molecule of nitrosoalkene **6** to afford functionalized indole **10**, which was isolated in 57% yield (overall yield from **5**). The reduction of the alkylated oxime **10** was achieved using an excess of metallic zinc/aqueous formic acid in tetrahydrofuran at room temperature for 24 h, affording the novel tryptamine **11** an 81% yield (Scheme 2).

**Scheme 3:**
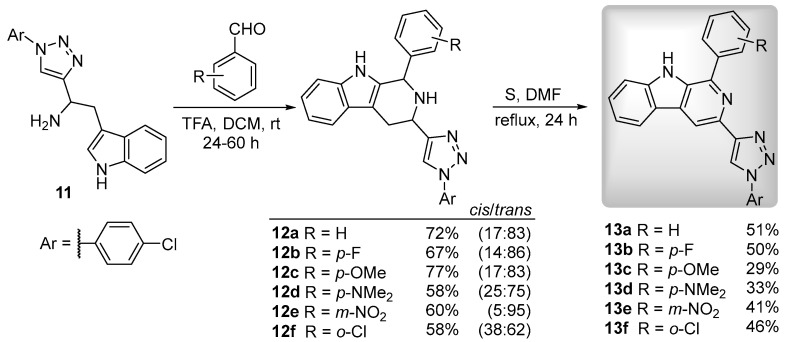
Synthesis of 3-triazolyl-β-carbolines **13a–f**.

Tryptophan analog **11** was used in the synthesis of 3-triazolyl-tetrahydro-β-carbolines **12** via the Pictet–Spengler approach. Trifluoroacetic acid-catalyzed condensation of tryptamine **11** with aromatic aldehydes (benzaldehyde, *p*-fluorobenzaldehyde, *p*-methoxybenzaldehyde, *p*-dimethylaminobenzaldehyde, *m*-nitrobenzaldehyde, *o*-chlorobenzaldehyde), carried out in dry dichloromethane for 24-60 h at room temperature, afforded the corresponding 3-triazolyl-tetrahydro-β-carbolines **12** in good yields (36–77%), isolated as mixtures of cis/trans isomers. The isomeric ratios were determined through NMR analysis of the isolated mixtures and the stereochemistry established by comparison with data previously reported for other β-carboline derivatives [8]. These 3-triazolyl-tetrahydro-β-carbolines underwent oxidation upon treatment with sulfur in DMF at reflux for 24 h, giving the target β-carbolines **13a–f** in moderate to good yields (37-52%) (Scheme 3).

### 2.2. Anticancer Activity

**Figure 3:**
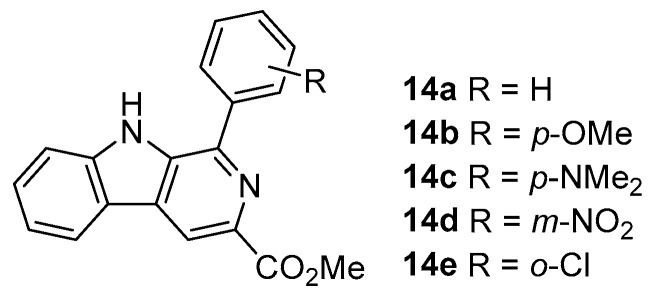
Structures of the studied methyl β-carboline-3-carboxylates **14a–e**.

The synthesized 3-triazolyl-β-carbolines **13a–f** and 3-tetrazolyl-β-carbolines **1a–c** [8] were evaluated for their anticancer activity in several colon cancer cells. In order to better evaluate and establish structure–activity relationships, methyl β-carboline-3-carboxylates **14a–e** were also synthesized from *L*-tryptophan methyl ester following a previously reported procedure, and their evaluation as colorectal anticancer agents was carried out (Figure 3) [50].

Impairment of the tumor suppressor p53 protein pathway is a critical event in cancer. Therefore, re-establishing p53 activity has become one of the most appealing therapeutic anticancer strategies [51,52,53]. Moreover, some β-carboline derivatives are known for their capability to activate the p53 activity [54,55]. In this context, we set out to determine whether the anticancer activity of the synthesized β-carbolines could involve selective activation of the p53 pathway. For that, the antiproliferative activity of β-carbolines **1a–c**, **13a–f**, and **14a–e** was evaluated against the human colorectal carcinoma HCT116 cell line expressing *wild type* (wt) p53, as well as the respective p53 knockout HCT116 derivative. The comparison of the activity of the compounds was made by analyzing the corresponding IC_50_ values calculated from the dose–response curves (Table 1).

In this initial screening, it was possible to observe that, despite our initial assumptions, the 3-triazolyl-β-carboline derivatives **13a–f** were not active against the studied cancer cell lines, with IC_50_ values ranging from 32 µM to values higher than 50 µM. Furthermore, among the methyl β-carboline-3-carboxylates, β-carboline **14c** was the one that presented the most promising results, with IC_50_ values of 15 µM and 18 µM against p53^+/+^ and p53^−/−^ HCT116 cancer cells. The remaining methyl β-carboline-3-carboxylates were not active (IC_50_ > 50 µM).

The 3-(1*H*-tetrazol-5-yl)-β-carbolines **1a**-**c** proved to be the most active compounds, with values of IC_50_ ranging from 3.3 µM to 4.6 µM against both cancer cell lines studied. It should be highlighted that the replacement of the methyl ester group of β-carbolines **14a** and **14b** by the bioisosteric benzyl-tetrazole group, leading to 3-(1*H*-tetrazol-5-yl)-β-carbolines **1a** and **1b**, respectively, resulted in a huge increase in anticancer activity. However, the results allowed us to conclude that none of the tested 3-(1*H*-tetrazol-5-yl)-β-carbolines selectively activate the p53 pathway.

The study was extended to evaluation of the anticancer activity of the 3-triazolyl- and 3-tetrazolyl-β-carbolines (**13a–f** and **1a–c**) against other human colorectal adenocarcinoma cell lines (SW837 and HT29), as well as the cytotoxicity against a normal human colon cell line (CCD-18Co), to determine the selectivity of these derivatives to cancer cells. The results of this study are summarized in Table 2.

From this study, we could confirm that the introduction of a triazole moiety directly attached to the β-carboline core has a negative impact on anticancer activity. In fact, it was observed that the 3-triazolyl-β-carboline derivatives **13a–f** were not active against the SW837 and HT29 cancer cell lines, nor did they show cytotoxicity against normal colon cell lines.

Interestingly, a different activity profile was observed with the 3-tetrazolyl-β-carboline derivatives **1a–c**. These β-carbolines proved to be active against the HT29 cells, showing IC_50_ values ranging from 5.9 to 9.6 µM. However, moderate activity was observed against the SW837 cells (IC_50_ from 29 µM to 44 µM).

**Figure 4:**
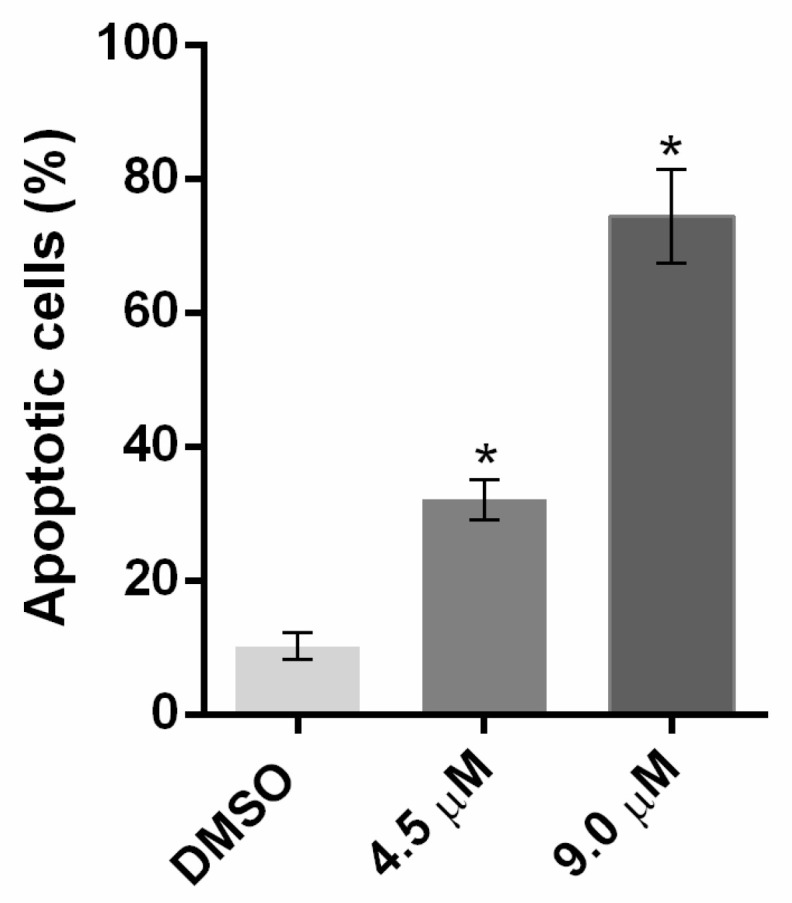
Effect of compound **1a** on apoptosis in HCT116 after 48 h of treatment; the percentage of Annexin-positive cells was analyzed by flow cytometry using PI and Annexin-V staining; data are mean ± SEM of 2 independent experiments. Values are significantly different from DMSO (* *p* < 0.05, unpaired Student’s *t*-test).

The study of the cytotoxicity against a normal colon cell line (CCD-18Co) revealed that 3-tetrazolyl-β-carboline **1a** has the highest selectivity for cancer cells, showing an IC_50_ value of 26 µM in these cells. In fact, this selectivity of compound **1a** to cancer cells was further evidenced in the normal human foreskin fibroblasts (HFF-1) cell line, in which compound **1a** presented an IC_50_ value of 29 μM (Table 3). Moreover, the results showed that the antiproliferative activity of compound **1a** was associated with apoptotic cell death (Figure 4).

The exact mechanism of action for these molecules was not studied; however, the selectivity of these compounds against cancer cells may be explained by the ability of β-carboline derivatives to bind to the DNA by intercalation. In fact, the DNA intercalation of β-carboline derivatives is associated with structural damage to the DNA of cancer cells, as well as with an inhibition of the DNA repair mechanism, inducing apoptosis [18,56,57].

Taking this into account, the anticancer activity of compound **1a** was further analyzed by testing its antiproliferative activity against distinct cell lines from distinct tumor tissues. As observed in Table 3, compound **1a** showed IC_50_ values below 8 μM against pancreatic carcinoma PANC-1, melanoma A375, hepatocarcinoma HEPG2, and breast adenocarcinoma MCF-7 cell lines. These results indicate that compound **1a** may be highly effective against distinct cancer types.

## 3. Experimental Section

### 3.1. Chemistry

#### 3.1.1. General Information

NMR spectra were recorded on a Bruker Avance III instrument, operating at 400 MHz (^1^H) or 100 MHz (^13^C). Chemical shifts are expressed in ppm relative to tetramethylsilane (TMS), and coupling constants (*J*) are in Hz. Infrared spectra (IR) were recorded using a Fourier Transform spectrometer coupled with a diamond Attenuated Total Reflectance (ATR) sampling accessory. High-resolution mass spectra (HRMS) were obtained on a TOF VG Autospect M spectrometer with electrospray ionization (ESI). Melting points were recorded in open glass capillaries. Thin Layer Chromatography (TLC) was performed using precoated silica gel plates. Flash chromatography was performed using silica gel 60 as a stationary phase. 3-Tetrazolyl-β-carbolines **1** [8], 3-triazolyl-α-bromooxime **10** [8], and β-carboline-3-carboxylates **14** [50] were prepared as described in the literature.

#### 3.1.2. 1-(1-(*p*-Chlorophenyl)-1*H*-1,2,3-triazol-4-yl)-2-(1*H*-indol-3-yl)ethanamine (**11**)

Oxime **10** (1.25 g, 2.13 mmol) was dissolved in the smallest amount of THF, 70% aqueous formic acid (40 mL) was added, and the solution was cooled to 0°C. Then, zinc powder (4.18 g, 63.9 mmol) was added portion-wise over 30 min. The reaction mixture was stirred for 24 h at room temperature. After this time, the mixture was filtered on a Celite pad and the celite was washed with ethyl acetate (3 × 15 mL). The filtrate was neutralized with a concentrated ammonia solution to pH 8, and then extracted with ethyl acetate (3 × 40 mL). The combined organic phases were washed with water, then dried over anhydrous Na_2_SO_4_, and the solvent evaporated off. The product was purified by flash chromatography [dichloromethane/methanol (90:10)], giving amine **11** as a brown oil (0.58 g, 81%). IR (ATR) 738, 828, 989, 1041, 1093, 1230, 1499, 1726, 2918, 3056, and 3141 cm^−1^. ^1^H NMR (CDCl_3_) δ: 3.10 (dd, *J* = 14.4 and 8.8 Hz, 1H), 3.38 (dd, *J* = 14.4 and 5.22 Hz, 1H), 3.42 (s, 1H), 4.53 (dd, *J* = 8.4 and 5.2 Hz, 1H), 6.98 (br s, 1H), 7.01–7.05 (m, 1H), 7.10–7.14 (m, 1H), 7.30 (br d, *J* = 8.0 Hz, 1H), 7.35-7.39 (m, 2H), 7.48-7.52 (m, 3H), 7.74 (s, 1H), 8.93 (s, 1H). ^13^C NMR (CDCl_3_) δ: 34.3, 48.9, 111.2, 112.2, 118.8, 118.9, 119.6, 121.6, 122.2, 123.1, 127.6, 129.9, 134.3, 135.7, 136.3, 153.3. HRMS (ESI-TOF) *m/z* for C_18_H_17_ClN_5_ [M+H^+^] calculated 338.1167, and found 338.1162.

^1^H NMR spectrum and ^13^C NMR spectrum of amine **11** are shown in Figure S1 and S2.

#### 3.1.3. General Procedure for the Synthesis of Tetrahydro-β-carbolines **12**

Trifluoroacetic acid (2 equiv.) was added to a solution of amine **11** (1 equiv.) and the appropriate aldehyde (1 equiv.) to dry dichloromethane (8 mL/mmol). The reaction mixture was stirred at room temperature, monitored by TLC, until all of the amine **11** was consumed. Upon completion, the reaction mixture was concentrated under reduced pressure, and the residue was dissolved in ethyl acetate (7 mL) and neutralized with an aqueous solution of Na_2_CO_3_ 10%. The resulting solution was extracted with ethyl acetate (3 × 25 mL), dried over anhydrous Na_2_SO_4_, and the solvent evaporated off. The products were purified by recrystallization in MeOH and obtained as a mixture of cis/trans isomers, unless otherwise stated.

##### 3-(1-(*p*-Chlorophenyl)-1*H*-1,2,3-triazol-4-yl)-1-phenyl-tetrahydro-β-carboline (**12a**)

This was obtained from the reaction of amine **11** (72 mg, 0.21 mmol) with benzaldehyde (22 μL, 0.21 mmol), as described in the general procedure (reaction time: 28 h), as a mixture of cis/trans isomers [65 mg, 71%, (17:83)]. *Major component* (*trans*-**12a**): IR (ATR): 748, 828, 1049, 1222, 1499 and 3398 cm^−1^. ^1^H NMR (Acetone-*d_6_*) δ: 3.10 (ddd, *J* = 14.8, 10.8 and 2.4 Hz, 1H), 3.30 (ddd, *J* = 15.0, 4.0 and 2.0 Hz, 1H), 4.54 (dd, *J* = 10.8 and 3.6 Hz, 1H), 5.46 (s, 1H), 7.00–7.05 (m, 2H), 7.26–7.28 (m, 1H), 7.31–7.35 (m, 3H), 7.44–7.47 (m, 2H), 7.50–7.52 (m, 1H), 7.61–7.64 (m, 2H), 7.96–8.00 (m, 2H), 8.60 (s, 1H), 9.53 (br s, 1H). HRMS (ESI-TOF) *m/z* for C_25_H_21_ClN_5_ [M+H^+^] calculated 426.1480, and found 426.1473.

##### 3-(1-(*p*-Chlorophenyl)-1*H*-1,2,3-triazol-4-yl)-1-(*p*-fluorophenyl)-tetrahydro-β-carboline (**12b**)

This was obtained from the reaction of amine **11** (73 mg, 0.22 mmol) with *p*-fluorobenzaldehyde (24 μL, 0.22 mmol), as described in the general procedure (reaction time: 40 h), as a mixture of cis/trans isomers [61 mg, 63%, (14:86)]. *Major component* (*trans*-**12b**): IR (ATR): 749, 832, 1137, 1204, 1500, 1655 and 3456 cm^−1^. ^1^H NMR (Acetone-*d_6_*) δ: 3.06–3.14 (m, 1H), 3.29 (ddd, *J* = 14.8, 4.0 and 1.6 Hz, 1H), 4.54 (dd, *J* = 10.8 and 4.0 Hz, 1H), 5.49 (s, 1H), 7.00–7.13 (m, 4H), 7.25–7.28 (m, 1H), 7.47–7.52 (m, 3H), 7.61–7.65 (m, 2H), 7.96–8.00 (m, 2H), 8.60 (s, 1H), 9.55 (br s, 1H). HRMS (ESI-TOF) *m/z* for C_25_H_20_ClFN_5_ [M+H^+^] calculated 444.1386, and found 444.1389.

##### 3-(1-(*p*-Chlorophenyl)-1*H*-1,2,3-triazol-4-yl)-1-(*p*-methoxyphenyl)-tetrahydro-β-carboline (**12c**)

This was obtained from the reaction of amine **11** (44 mg, 0.13 mmol) with *p*-methoxybenzaldehyde (15 μL, 0.13 mmol), as described in the general procedure (reaction time: 40 h). The product was purified by flash chromatography [ethyl acetate/hexane (1:1)] and obtained as a mixture of cis/trans isomers [45 mg, 77%, (12:88)]. *Major component* (*trans*-**12c**): IR (ATR): 746, 830, 1136, 1183, 1203, 1502, 1672 and 2839 cm^−1^. ^1^H NMR (Acetone-*d_6_*) δ: 3.09 (ddd, *J* = 14.8, 10.8 and 2.4 Hz, 1H), 3.29 (ddd, *J* = 14.8, 3.6 and 1.6 Hz, 1H), 3.79 (s, 3H), 4.53 (dd, *J* = 11.2 and 3.6 Hz, 1H), 5.41 (s, 1H), 6.88–6.92 (m, 2H), 6.99–7.05 (m, 2H), 7.27–7.29 (m, 1H), 7.33–7.37 (m, 2H), 7.49–7.52 (m, 1H), 7.60–7.64 (m, 2H), 7.95–7.99 (m, 2H), 8.58 (s, 1H), 9.47 (s, 1H). HRMS (EI-TOF) *m/z* for C_26_H_23_ClN_5_O [M+H^+^] calculated 456.1586, and found 456.1580.

##### 3-(1-(*p*-Chlorophenyl)-1*H*-1,2,3-triazol-4-yl)-1-(*p*-dimethylaminophenyl)-tetrahydro-β-carboline (**12d**)

This was obtained from the reaction of amine **11** (89 mg, 0.26 mmol) with *p*-(dimethylamino)benzaldehyde (39 mg, 0.26 mmol), as described in the general procedure (reaction time: 58 h). The product was purified by flash chromatography [ethyl acetate/hexane (1:1)] and obtained as a mixture of cis/trans isomers [43 mg, 36%, (30:70)]. *Major component* (*trans*-**12d**): IR (ATR): 739, 829, 1035, 1094, 1438, 1500, 1654 cm^−1^. ^1^H NMR (Acetone-*d_6_*) δ: 2.92 (s, 6H), 3.05–3.09 (m, 1H), 3.27–3.32 (m, 1H), 4.50–4.53 (m, 1H), 5.32 (s, 1H), 6.67–6.73 (m, 2H), 7.02–7.07 (m, 2H), 7.22–7.25 (m, 2H), 7.29–7.32 (m, 1H), 7.50–7.53 (m, 1H), 7.59–7.63 (m, 2H), 7.93–7.97 (m, 2H), 8.55 (s, 1H), 9.46 (s, 1H). HRMS (ESI-TOF) *m/z* for C_27_H_26_ClN_6_ [M+H^+^] calculated 469.1902, and found 469.1898.

##### 3-(1-(*p*-Chlorophenyl)-1*H*-1,2,3-triazol-4-yl)-1-(*m*-nitrophenyl)-tetrahydro-β-carboline (**12e**)

This was obtained from the reaction of amine **11** (79 mg, 0.24 mmol) with *m*-nitrobenzaldehyde (36 mg, 0.24 mmol), as described in the general procedure (reaction time: 28 h), as a mixture of cis/trans isomers [67 mg, 61%, (5:95)]. *Major component* (*trans*-**12e**): IR (ATR): 747, 829, 1029, 1094, 1229, 1500 and 3307 cm^−1^. ^1^H NMR (Acetone-*d_6_*) δ: 3.16 (ddd, *J* = 14.8, 10.8 and 2.4 Hz, 1H), 3.30 (ddd, *J* = 15.2, 4.0 and 2.0 Hz, 1H), 4.60 (dd, *J* = 10.8 and 4.0 Hz, 1H), 5.69 (s, 1H), 7.01–7.08 (m, 2H), 7.23–7.25 (m, 1H), 7.52–7.54 (m, 1H), 7.62–7.67 (m, 3H), 7.93–7.99 (m, 3H), 8.19 (ddd, *J* = 8.4, 2.4 and 1.2 Hz, 1H), 8.35–8.36 (m, 1H), 8.65 (s, 1H), 9.69 (br s, 1H). ^13^C NMR (Acetone-*d_6_*) δ: 52.4, 59.4, 109.7, 112.0, 118.8, 119.8, 120.4, 122.2, 122.5, 123.6, 124.5, 128.1, 130.6, 130.7, 134.3, 135.4, 136.3, 137.1, 137.9, 145.9, 149.3, 152.5. HRMS (ESI-TOF) *m/z* for C_25_H_20_ClN_6_O_2_ [M+H^+^] calculated 471.1331, and found 471.1323.

##### 3-(1-(*p*-Chlorophenyl)-1*H*-1,2,3-triazol-4-yl)-1-(*o*-chlorophenyl)-tetrahydro-β-carboline (**12f**)

This was obtained from the reaction of amine **11** (133 mg, 0.39 mmol) with *o*-chlorobenzaldehyde (44 μL, 0.39 mmol), as described in the general procedure (reaction time: 40 h). The product was purified by flash chromatography [ethyl acetate/hexane (1:2)] and obtained as a mixture of cis/trans isomers [70 mg, 39%, (50:50)]. *Component trans*-**12f**: IR (ATR): 739, 830, 989, 1036, 1095, 1438, 1500, 2858 and 3281 cm^−1^. ^1^H NMR (Acetone-*d_6_*) δ 3.09–3.17 (m, 1H), 3.29–3.36 (m, 1H), 4.57 (dd, *J* = 10.6 and 4.0 Hz, 1H), 5.99 (s, 1H), 6.91 (dd, *J* = 8.0 and 2.0 Hz, 1H), 7.02–7.20 (m, 2H), 7.22–7.31 (m, 2H), 7.34–7.47 (m, 2H), 7.52–7.59 (m, 3H), 7.90–7.94 (m, 2H), 8.55 (s, 1H), 9.66 (s, 1H). HRMS (ESI-TOF) *m/z* for C_25_H_20_Cl_2_N_5_ [M+H^+^] calculated 460.1090, and found 460.1082.

^1^H NMR spectrum and ^13^C NMR spectrum of tetrahydro-β-carbolines **12a-f** are shown in Figures S3–S14.

#### 3.1.4. General Procedure for the Synthesis of β-Carbolines **13**

Sulfur powder (3 equiv.) was added to a solution of the appropriate tetrahydro-β-carboline **12** (1 equiv.) in dimethylformamide (20 mL/mmol). The reaction mixture was stirred under reflux for 24 h. After this time, the solvent was evaporated off. The product was precipitated by addition of diethyl ether, then filtered and dried under vacuum to obtain the products in a pure form, unless otherwise stated.

##### 3-(1-(*p*-Chlorophenyl)-1*H*-1,2,3-triazol-4-yl)-1-phenyl-β-carboline (**13a**)

This was obtained from tetrahydro-β-carboline **12a** (81 mg, 0.19 mmol) as a dark yellow solid (28 mg, 51%). mp 207.5–207.9 °C (from diethyl ether). IR (ATR): 738, 826, 1027, 1098, 1496, 1630 and 1670 cm^−1^. ^1^H NMR (DMSO-*d_6_*) δ: 7.29–7.33 (m, 1H), 7.56–7.61 (m, 2H), 7.65–7.72 (m, 5H), 8.12–8.18 (m, 4H), 8.42 (d, *J* = 8.0 Hz, 1H), 8.88 (s, 1H), 9.31 (s, 1H), 11.69 (s, 1H). ^13^C NMR (DMSO-*d_6_*) δ: 110.3, 112.6, 119.9, 120.3, 121.0, 121.8, 122.0, 128.6, 128.7, 128.8, 128.9, 129.8, 130.3, 132.6, 132.9, 135.6, 137.7, 138.7, 141.7, 141.8, 149.5. HRMS (ESI-TOF) *m/z* for C_25_H_17_ClN_5_ [M+H^+^] calculated 422.1167, and found 422.1164.

##### 3-(1-(*p*-Chlorophenyl)-1*H*-1,2,3-triazol-4-yl)-1-(*p*-fluorophenyl)-β-carboline (**13b**)

This was obtained from tetrahydro-β-carboline **12b** (72 mg, 0.16 mmol) as a dark yellow solid (36 mg, 50%). mp 237.3–238.1°C (from diethyl ether). IR (ATR): 738, 829, 1037, 1094, 1199, 1401, 1498, 1630, 1655 and 3062 cm^−1^. ^1^H NMR (DMSO-*d_6_*) δ: 7.31 (t, *J* = 8.0 Hz, 1H), 7.46–7.52 (m, 2H), 7.58–7.62 (m, 1H), 7.67–7.72 (m, 3H), 8.12–8.16 (m, 2H), 8.21–8.24 (m, 2H), 8.43 (d, *J* = 8.0 Hz, 1H), 8.87 (s, 1H), 9.32 (s, 1H), 11.69 (s, 1H). ^13^C NMR (DMSO-*d_6_*) δ: 110.3, 112.6, 115.6 (d, *J* = 21.3 Hz), 120.0, 120.3, 121.0, 121.8, 122.0, 128.7, 129.8, 130.5, 130.9 (d, *J* = 8.4 Hz), 132.6, 132.9, 134.1 (d, *J* = 2.7 Hz), 135.5, 138.6, 140.7, 141.8, 149.3, 162.6 (d, *J* = 244.4 Hz). HRMS (ESI-TOF) *m/z* for C_25_H_16_ClFN_5_ [M+H^+^] calculated 440.1073, and found 440.1077.

##### 3-(1-(*p*-Chlorophenyl)-1*H*-1,2,3-triazol-4-yl)-1-(*p*-methoxyphenyl)-β-carboline (**13c**)

This was obtained from tetrahydro-β-carboline **12c** (71 mg, 0.16 mmol). The product was purified by flash chromatography [ethyl acetate/hexane (1:1)] and obtained as a brown solid (26 mg, 37%). mp 219.7-221.3°C (from diethyl ether). IR (ATR): 738, 826, 1023, 1093, 1170, 1498, 1607, 1628 and 3036 cm^−1^. ^1^H NMR (Acetone-*d_6_*) δ: 3.91 (s, 3H), 7.12–7.15 (m, 2H), 7.33 (ddd, *J* = 8.0, 6.8 and 0.8 Hz, 1H), 7.57 (ddd, *J* = 8.0, 6.8 and 1.2 Hz, 1H), 7.64–7.69 (m, 3H), 8.07–8.10 (m, 2H), 8.11–8.14 (m, 2H), 8.39 (d, *J* = 8.0 Hz, 1H), 8.84 (s, 1H), 9.04 (s, 1H), 10.78 (s, 1H). ^13^C NMR (Acetone-*d_6_*) δ: 55.8, 110.3, 113.2, 115.0, 120.4, 120.9, 122.6, 122.7, 122.8, 129.2, 130.7, 130.8, 131.4, 132.0, 133.8, 134.3, 137.1, 140.8, 142.6, 143.2, 151.4, 161.3. HRMS (ESI-TOF) *m/z* for C_26_H_19_OClN_5_ [M+H^+^] calculated 452.1273, and found 452.1269.

##### 3-(1-(*p*-Chlorophenyl)-1*H*-1,2,3-triazol-4-yl)-1-(*p*-dimethylaminophenyl)-β-carboline (**13d**)

This was obtained from tetrahydro-β-carboline **12d** (40 mg, 0.09 mmol) as an orange solid (20 mg, 48%). mp 244.2-245.6°C (from diethyl ether). IR (ATR): 747, 828, 1021, 1465, 1603 and 2800 cm^−1^. ^1^H NMR (DMSO-*d_6_*) δ: 2.85 and 3.08 (s, 6H), 6.90 and 7.02 (d, *J* = 8.0 Hz, 1H), 7.32-7.36 (m, 1H), 7.62-7.73 (m, 4H), 7.94–8.08 (m, 4H), 8.42 (d, *J* = 6.8 Hz, 1H), 8.87 and 8.88 (s, 1H), 9.39 and 9.42 (s, 1H), 11.94 and 12.00 (s, 1H). ^13^C NMR (DMSO-*d_6_*) δ: 34.4, 110.4, 112.3, 112.9, 120.4, 120.8, 121.2, 121.8, 122.3, 129.4, 129.9, 130.2, 130.5, 131.0, 132.0, 133.2, 135.3, 141.3, 142.6, 151.1. HRMS (ESI-TOF) *m/z* for C_27_H_22_ClN_6_ [M+H^+^] calculated 465.1589, and found 465.1584.

##### 3-(1-(*p*-Chlorophenyl)-1*H*-1,2,3-triazol-4-yl)-1-(*m*-nitrophenyl)-β-carboline (**13e**)

This was obtained from tetrahydro-β-carboline **12e** (33 mg, 0.07 mmol). The product was purified by flash chromatography [ethyl acetate/hexane (1:1)] and obtained as a yellow solid (17 mg, 52%). mp 237.3-239.1°C (from diethyl ether). IR (ATR): 737, 827, 1026, 1093, 1244, 1346, 1400, 1499 and 1526 cm^−1^. ^1^H NMR (DMSO-*d_6_*) δ: 7.33 (ddd, *J* = 7.6, 6.4 and 0.8 Hz, 1H), 7.61 (ddd, *J* = 7.6, 6.8 and 0.8 Hz, 1H), 7.66-7.72 (m, 3H), 7.94 (t, *J* = 8.0 Hz, 1H), 8.10–8.14 (m, 2H), 8.41 (ddd, *J* = 8.4, 2.4 and 0.8 Hz, 1H), 8.44 (d, *J* = 7.6 Hz, 1H), 8.57 (dt, *J* = 8.0 and 1.2 Hz, 1H), 8.85 (t, *J* = 1.6 Hz, 1H), 8.93 (s, 1H), 9.31 (s, 1H), 11.83 (s, 1H).^13^C NMR (DMSO-*d_6_*) δ: 111.1, 112.4, 120.1, 120.3, 121.0, 121.8, 122.1, 123.2, 123.4, 128.8, 129.8, 130.3, 130.7, 132.9, 132.9, 135.1, 135.5, 1329.2, 139.4, 139.4, 141.7, 148.2, 149.3. HRMS (ESI-TOF) *m/z* for C_25_H_16_O_2_ClN_6_ [M+H^+^] calculated 467.1018, and found 467.1017.

##### 3-(1-(*p*-Chlorophenyl)-1*H*-1,2,3-triazol-4-yl)-1-(*o*-chlorophenyl)-β-carboline (**13f**)

This was obtained from tetrahydro-β-carboline **12f** (45 mg, 0.10 mmol) as a dark yellow solid (18 mg, 40%). mp 219.7-221.3°C (from diethyl ether). IR (ATR): 738, 823, 1037, 1090, 1498, 1625 and 3070 cm^−1^. ^1^H NMR (DMSO-*d_6_*) δ: 7.27-7.33 (m, 1H), 7.57-7.73 (m, 8H), 8.11–8.14 (m, 2H), 8.43 (d, *J* = 8.0 Hz, 1H), 8.94 (s, 1H), 9.23 (s, 1H), 11.47 (s, 1H). ^13^C NMR (DMSO-*d_6_*) δ: 110.8, 112.2, 119.7, 120.0, 120.9, 121.7, 122.1, 127.4, 128.6, 129.2, 129.7, 129.8, 130.4, 132.0, 132.6, 132.8, 133.6, 135.5, 136.9, 138.6, 141.3, 141.5, 149.5. HRMS (ESI-TOF) *m/z* for C_25_H_16_Cl_2_N_5_ [M+H^+^] calculated 456.0777, and found 456.0776.

^1^H NMR and ^13^C NMR spectrum ofβ-carbolines **13a-f** are shown in Figures S15-S26.

### 3.2. Anticancer Activity

#### 3.2.1. Human Cell Lines

Human colorectal adenocarcinoma HCT116 cell lines expressing p53 and its p53-null isogenic derivative (HCT116 p53^−/−^) were provided by B. Vogelstein (The Johns Hopkins Kimmel Cancer Center, Baltimore, MD, USA). Human colorectal adenocarcinoma SW837, HT29, pancreatic carcinoma PANC-1, breast adenocarcinoma MCF-7, hepatocarcinoma HEPG2, melanoma A375, normal human foreskin fibroblasts HFF-1, and normal human colon CCD-18Co cell lines were purchased from ATCC (Rockville, MD, USA). The cancer cell lines were routinely cultured with RPMI-1640 medium from Biowest (HCT116, HCT116 p53^−/−^, SW837, HT29, A375, MCF-7, HEPG-2, and HFF-1) or DMEM with 5% glucose (HFF-1) and supplemented with 10% fetal bovine serum from Gibco (Alfagene, Lisboa, Portugal). All cells were maintained in a humidified incubator at 37 °C and 5% CO_2._

#### 3.2.2. Sulforhodamine B (SRB) Assay

Human cell lines were seeded in 96-well plates at a density of 5.0 × 10^3^ (HCT116 p53 ^−/−^, HCT116 wt, HT29, SW837, CCD-18Co, HFF-1, and MCF-7 cell lines) or 4.5 × 10^3^ (A375, HEPG2 and PANC-1 cell lines) cells per well, for 24 h. Cells were treated with the appropriate compound, with serial dilutions ranging from 0.2 to 50 µM, for 48 h. Following the incubation, the effects on cell proliferation were evaluated through the SRB assay as stated by Ramos et al. [58]. The IC_50_ values were determined using the software GraphPad Prism version 9.0.

#### 3.2.3. Annexin-V Assay

The analysis of apoptotic cell death was performed essentially as described by Ramos et al. [58]. Briefly, 1.5 × 10^5^ HCT116 cells/well were seeded in 6-well plates, allowed to adhere overnight, and then treated with 4.5 and 9.0 μM of compound **1a**. After 48 h of treatment, cells were stained using the Annexin V-FITC Apoptosis Detection Kit I from BD Biosciences (Enzifarma, Porto, Portugal), according to the manufacturer’s instructions. The AccuriTM C6 flow cytometer and the BD Accuri C6 software (BD Biosciences) were used.

## 4. Conclusions

The synthesis of β-carboline derivatives and their activity against several human colorectal adenocarcinoma cell lines has been disclosed. A synthetic route to novel 3-(1,2,3-triazol-4-yl)-β-carboline derivatives was established via the Pictet–Spengler approach using a tryptophan analog, where the carboxylic acid was replaced by a triazole moiety. In order to better evaluate and establish structure–activity relationships, methyl β-carboline-3-carboxylates and 3-tetrazolyl-β-carbolines were also synthesized from the corresponding tryptamine analogs.

The antiproliferative activity of the synthesized β-carbolines against colorectal cancer cells revealed that the 3-(1*H*-tetrazol-5-yl)-β-carbolines, particularly compound **1a**, were the most active molecules, and that they act through a p53-independent apoptotic pathway. Moreover, compound **1a** demonstrated a high selectivity to cancer cells, which may be attributed to the DNA intercalating ability of β-carbolines, with subsequent inhibition of DNA repair mechanisms, in cancer cell lines.

The disclosed results also indicate that the presence of a triazole moiety at β-carboline’s C-3 carbon severely hinders the overall biological activity of these heterocycles, while a tetrazole moiety at the same position greatly increases the cytotoxicity against human colon cancer cell lines when compared with the methyl ester β-carboline derivatives.

Further studies on the 3-tetrazolyl-β-carboline derivative, with the highest selectivity for cancer cells, unveiled an interesting anticancer profile by targeting cancer cells from distinct tissues.

This study, therefore, unveils a promising anticancer agent worthy of being explored in future works.

## Figures and Tables

**Table 1:** Antiproliferative activity of β-carbolines **13a–f**, **1a–c,** and **14a–e** against colorectal HCT116 carcinoma cells with and without (HCT116 p53^−/−^) wt p53.

	IC_50_ (μM)	
β-Carboline	HCT116	HCT116 p53^−/−^
**Etoposide**	0.6 ± 0.05	0.9 ± 0.07
**13a**	39 ± 2.16	43 ± 0.82
**13b**	>50	>50
**13c**	32 ± 2.45	>50
**13d**	>50	>50
**13e**	>50	>50
**13f**	>50	>50
**1a**	4.5 ± 0.05	4.6 ± 0.43
**1b**	4.3 ± 0.08	4.2 ± 0.31
**1c**	3.4 ± 0.16	3.3 ± 0.73
**14a**	>50	>50
**14b**	>50	>50
**14c**	15 ± 1.41	18 ± 0.47
**14d**	>50	>50
**14e**	>50	>50

IC_50_ values were determined after 48 h of treatment (growth obtained with vehicle was set as 100%). Data are mean ± SEM of three to five independent experiments.

**Table 2:** Antiproliferative activity of β-carbolines **13a**-**f** and **1a**-**c** against colorectal adenocarcinoma and normal colon cell lines.

	IC_50_ (μM)		
β-Carboline	SW837	HT29	CCD-18Co
**Etoposide**	0.9 ± 0.07	0.7 ± 0.1	0.5 ± 0.07
**13a**	>50	>50	>50
**13b**	>50	>50	>50
**13c**	>50	>50	>50
**13d**	>50	>50	>50
**13e**	>50	>50	>50
**13f**	>50	>50	>50
**1a**	44 ± 0.82	9.0 ± 0.55	26 ± 0.82
**1b**	25 ± 1.63	5.9 ± 1.19	3.4 ± 0.29
**1c**	29 ± 3.74	9.6 ± 1.18	7.5 ± 0.16

IC_50_ values were determined after 48 h of treatment (growth obtained with vehicle was set as 100%). Data are mean ± SEM of three to five independent experiments.

**Table 3:** Antiproliferative activity of β-carboline **1a** against pancreatic, melanoma, hepatocarcinoma, and breast cancer cells.

	IC_50_ (μM)				
**β-Carboline**	**HFF-1**	**PANC-1**	**A375**	**HEPG2**	**MCF-7**
**Etoposide**	1.1 ± 0.01	3.1 ± 0.05	0.9 ± 0.07	1.7 ± 0.2	2.1 ± 0.03
** 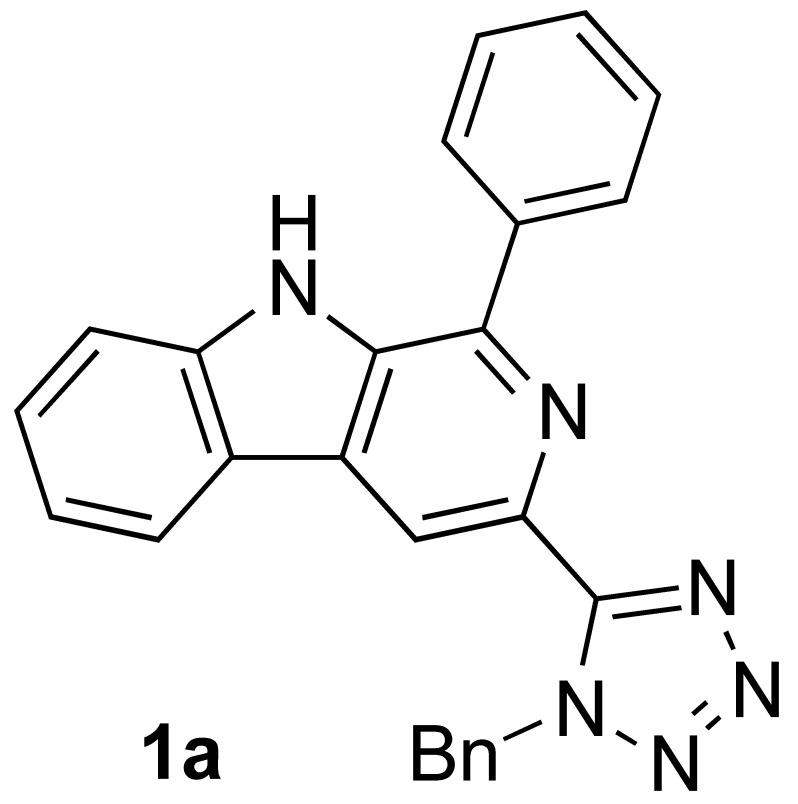 **	29 ± 1.50	4.0 ± 0.40	5.6 ± 0.20	7.5 ± 1.27	7.7 ± 0.70

IC_50_ values were determined after 48 h of treatment (growth obtained with vehicle was set as 100%). Data are mean ± SEM of three to five independent experiments.

## Data Availability

Not applicable.

## Supplementary Materials

The following supporting information can be downloaded at: https://www.mdpi.com/article/10.3390/ph15121510/s1, Figure S1: ^1^H NMR spectrum of amine **11,** Figure S2: ^13^C NMR spectrum of amine **11**. Figures S3–S14: ^1^H NMR and ^13^C NMR spectrum of tetrahydro-β-carbolines **12a-f**. Figures S15–S26: ^1^H NMR and ^13^C NMR spectrum of β-carbolines **13a-f**.
