# Supplementary material for: 3-(1,2,3-Triazol-4-yl)-β-Carbolines and 3-(1H-Tetrazol-5-yl)-β-Carbolines: Synthesis and Evaluation as Anticancer Agents
Source: Pharmaceuticals (Basel). 2022 Dec 3;15(12):1510. doi: 10.3390/ph15121510 (PMC9785278; doi:10.3390/ph15121510)
Supplement: Supplementary file 1 [file pharmaceuticals-15-01510-s001.zip › pharmaceuticals-2059126-supplementary.pdf]

3-(1,2,3-Triazol-4-yl)-β-Carbolines and 3-(1*H*-Tetrazol-5-yl)-β-Carbolines: Synthesis and Evaluation as Anticancer Agents

João L. P. Ribeiro,[1] Joana B. Loureiro [2], Susana M. M. Lopes,[1] Lucília Saraiva,[2] Teresa M. V. D. Pinho e Melo[1]*

[1]University of Coimbra, Coimbra Chemistry Centre-Institute of Molecular Sciences, and Department of Chemistry, 3004-535 Coimbra

[2]LAQV/REQUIMTE, Laboratory of Microbiology, Department of Biological Sciences, Faculty of Pharmacy, University of Porto, Rua Jorge Viterbo Ferreira, 228, 4050-313 Porto, Portugal

Supporting Information

Table of Contents

^{1}H and ^{13}C NMR spectra of all new compounds…………...…………………………… 2

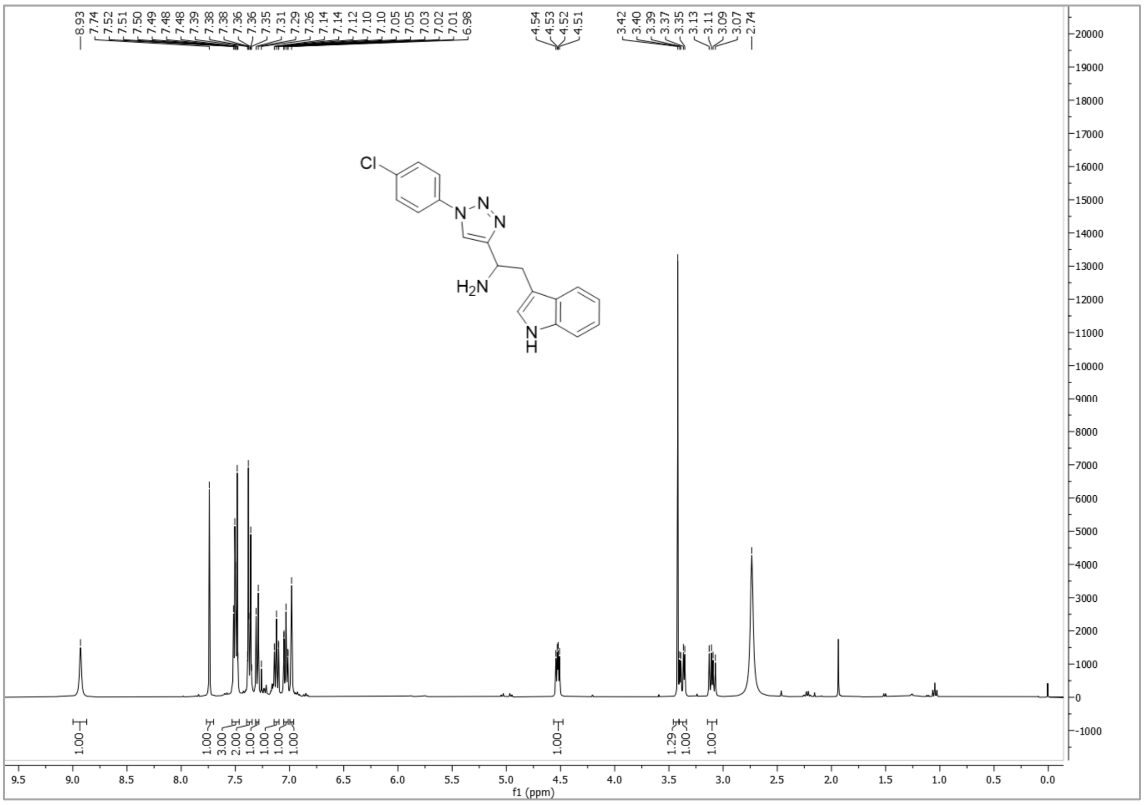

Figure S1. ^{1}H NMR spectrum of amine **11** (400 MHz, $CDCl_3$).

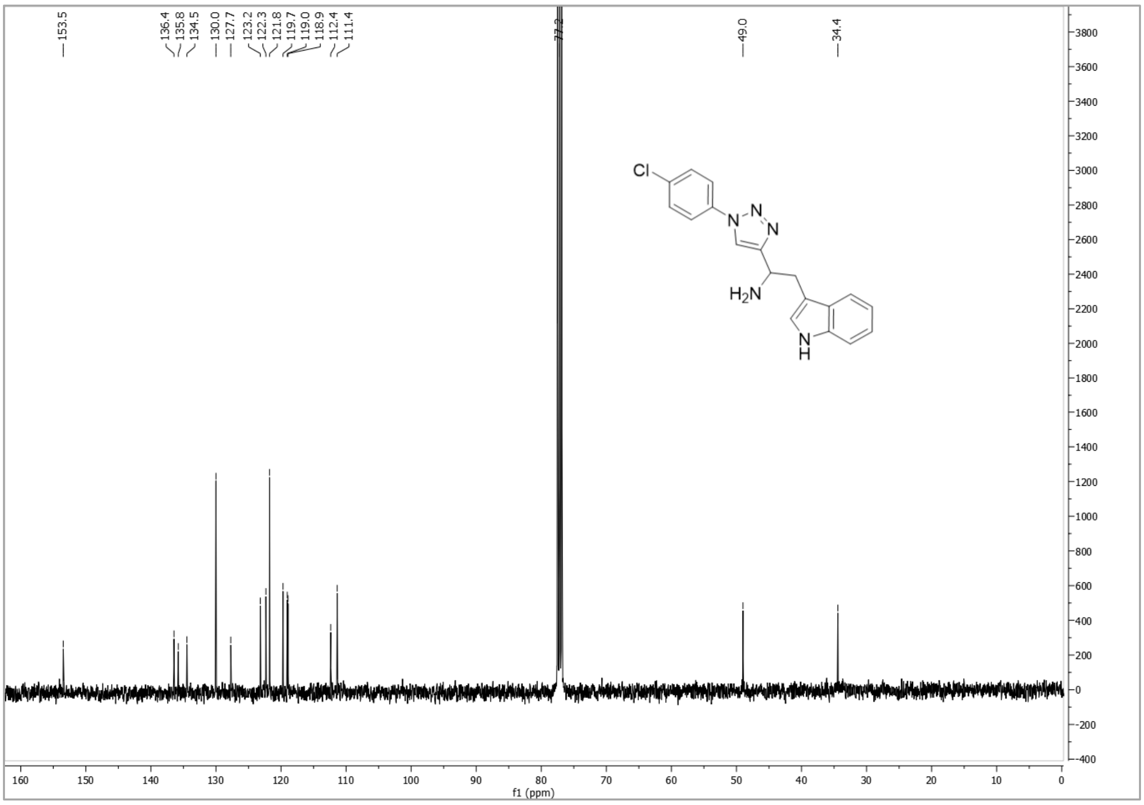

Figure S2. ^{13}C NMR spectrum of amine **11** (100 MHz, $CDCl_3$).

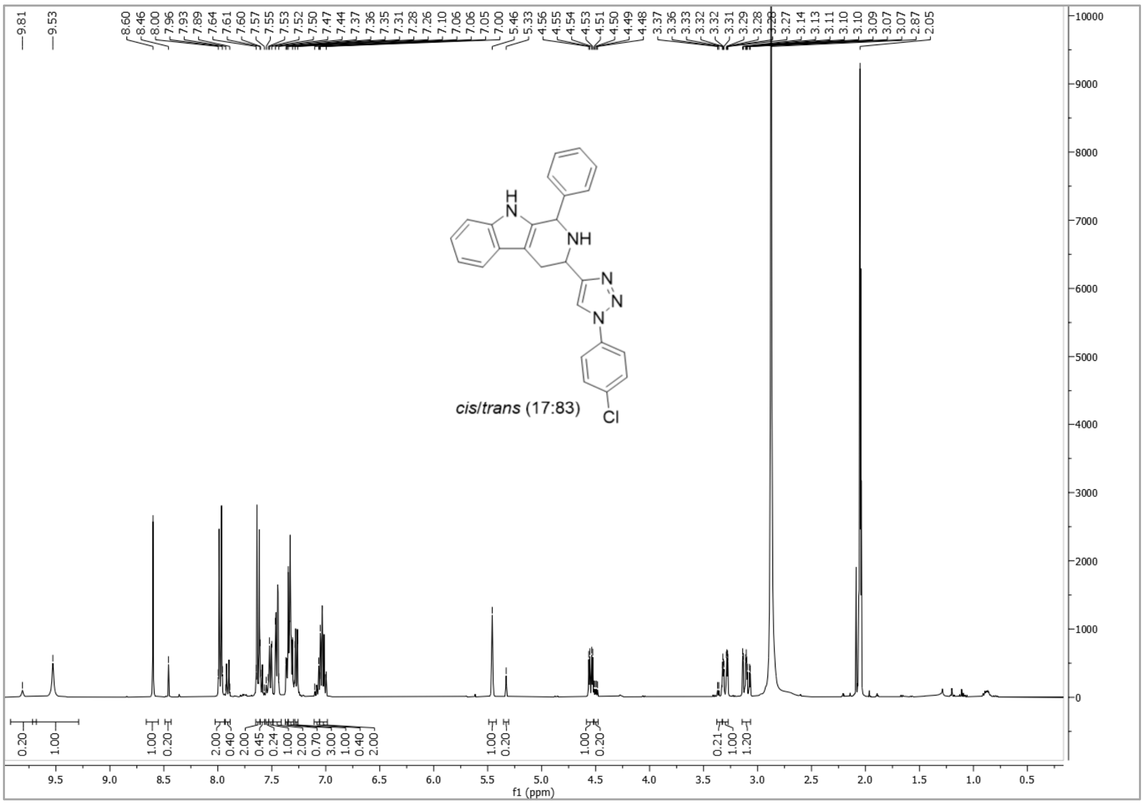

Figure S3. ^{1}H NMR spectrum of compounds **12a** (400 MHz, Acetone-d_6).

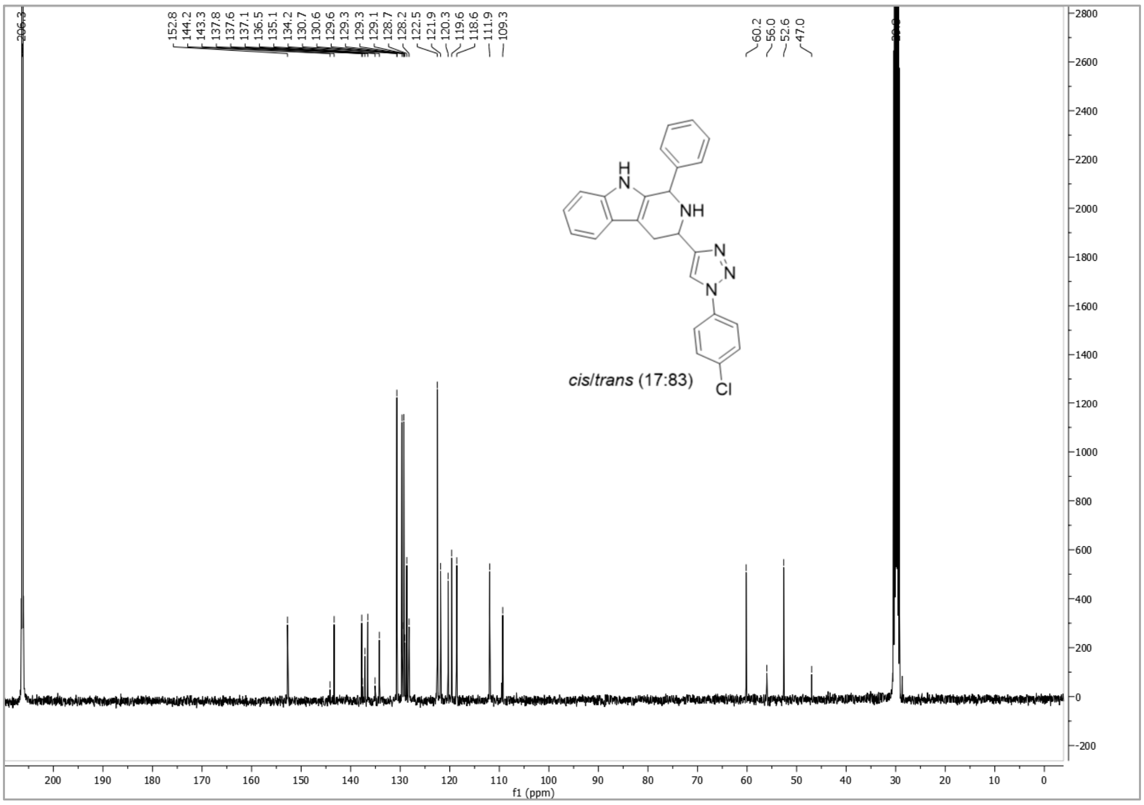

Figure S4. ^{13}C NMR spectrum of compounds **12a** (100 MHz, Acetone-d_6).

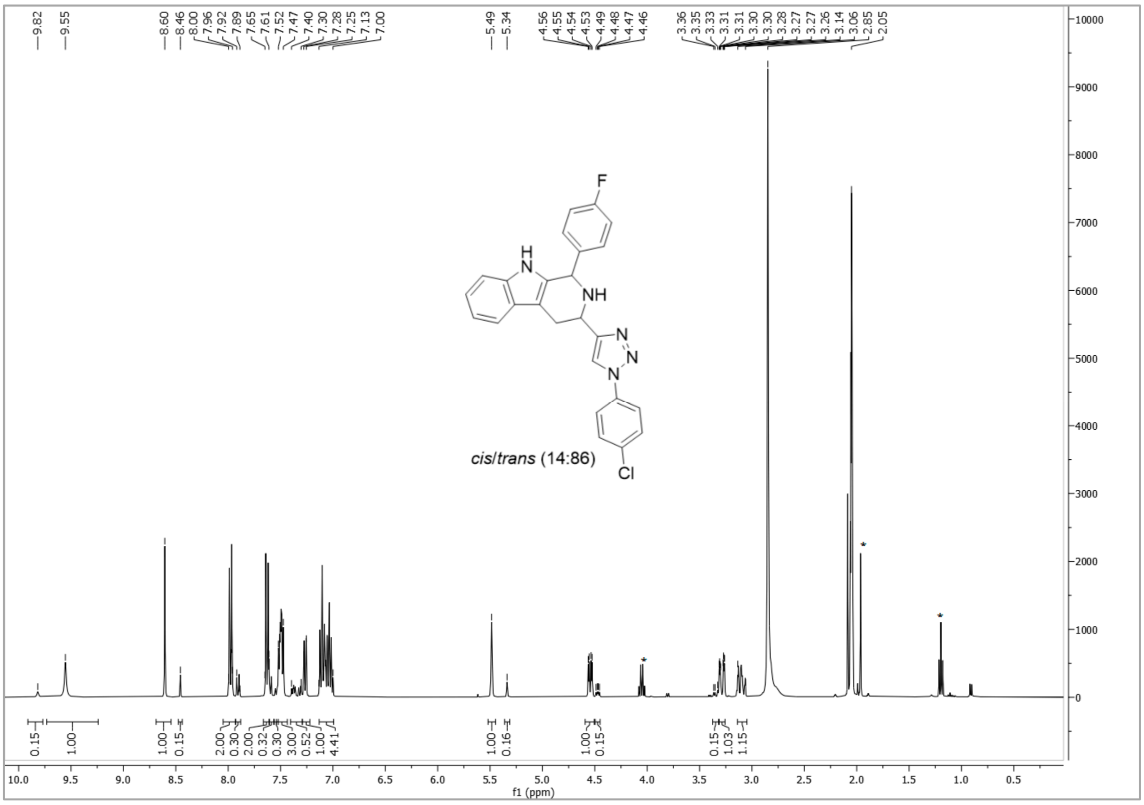

Figure S5. ^{1}H NMR spectrum of compounds **12b** (400 MHz, Acetone-d_6). *Solvent (EtOAc).

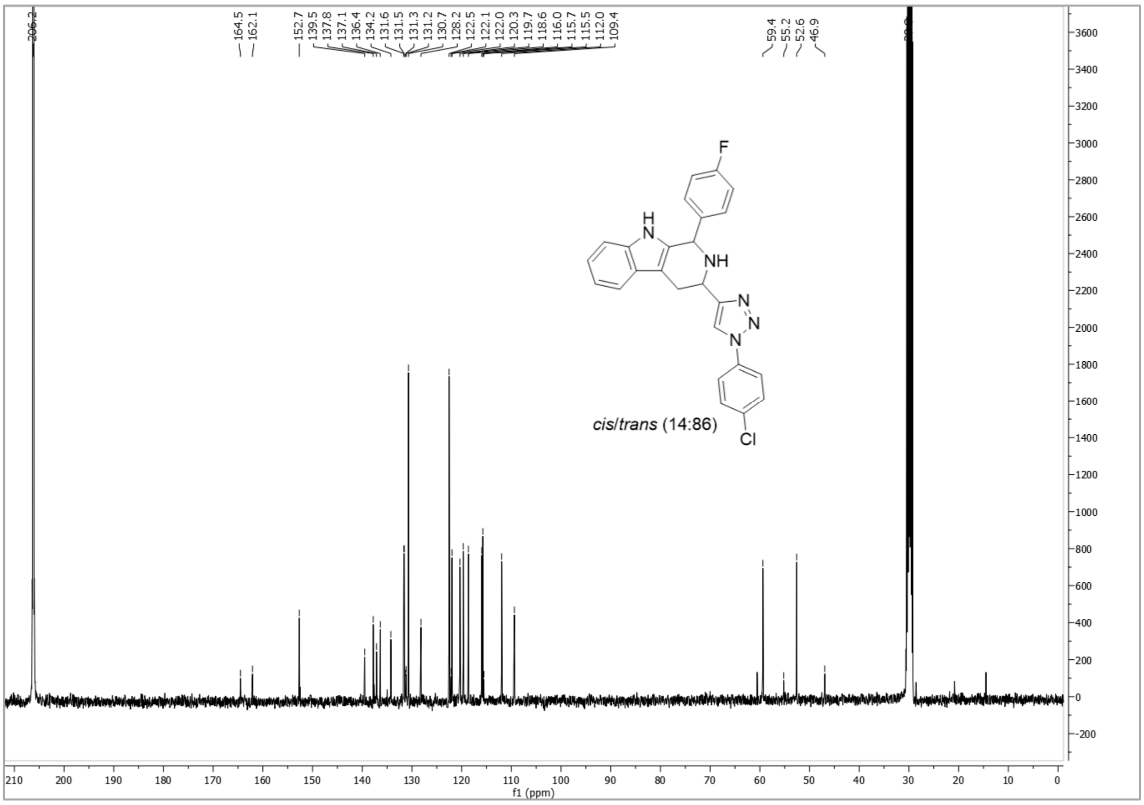

Figure S6. ^{13}C NMR spectrum of compounds **12b** (100 MHz, Acetone-d_6).

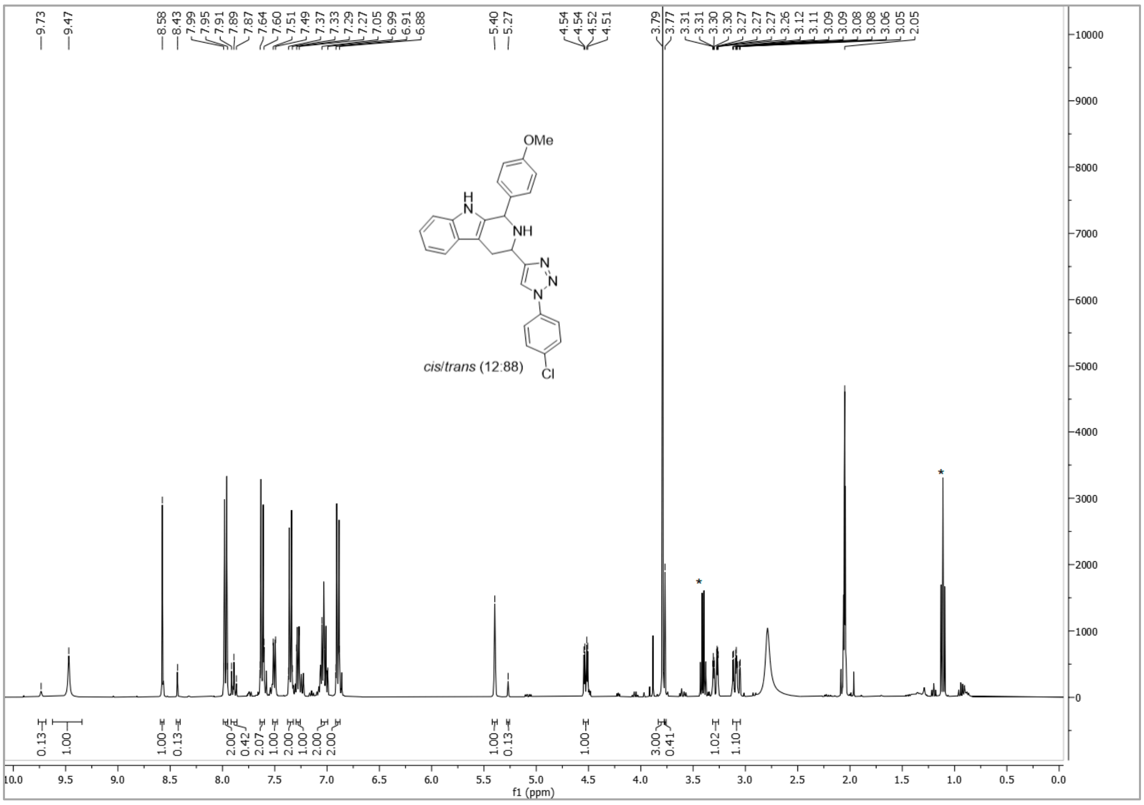

Figure S7. ^{1}H NMR spectrum of compounds **12c** (400 MHz, Acetone-d_6). *Solvent (Et_2O).

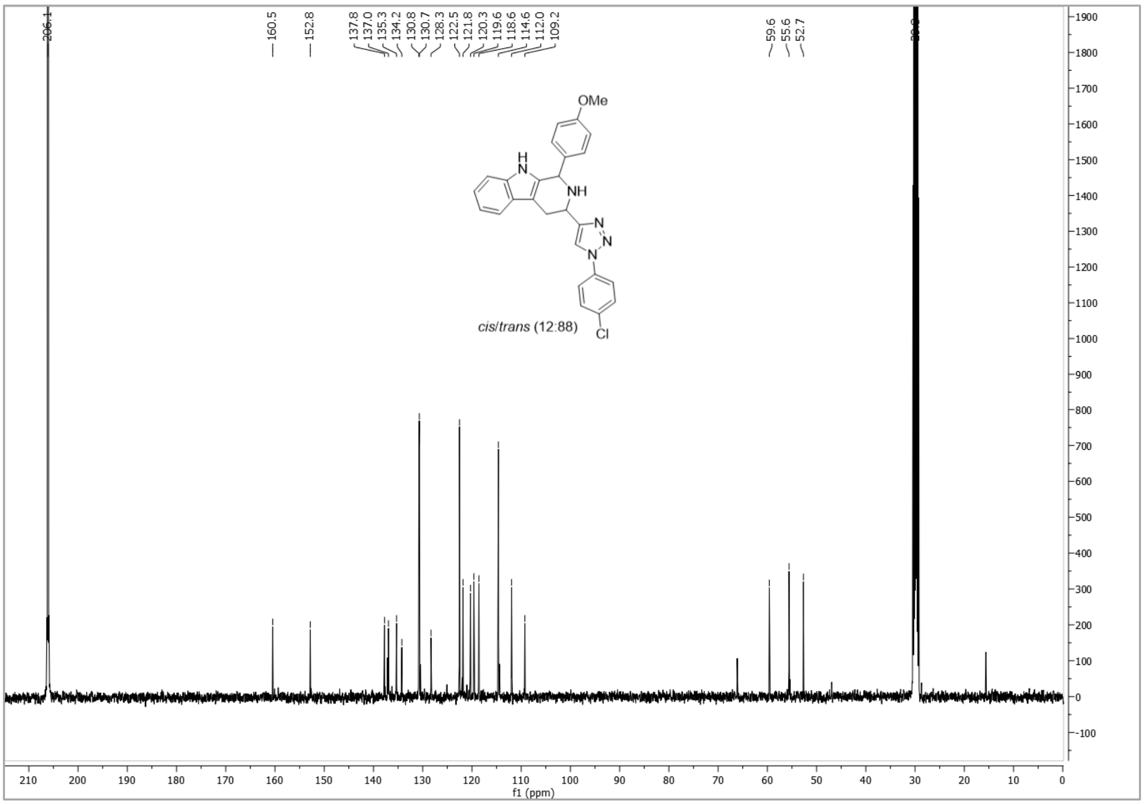

Figure S8. ^{13}C NMR spectrum of compounds **12c** (100 MHz, Acetone-d_6).

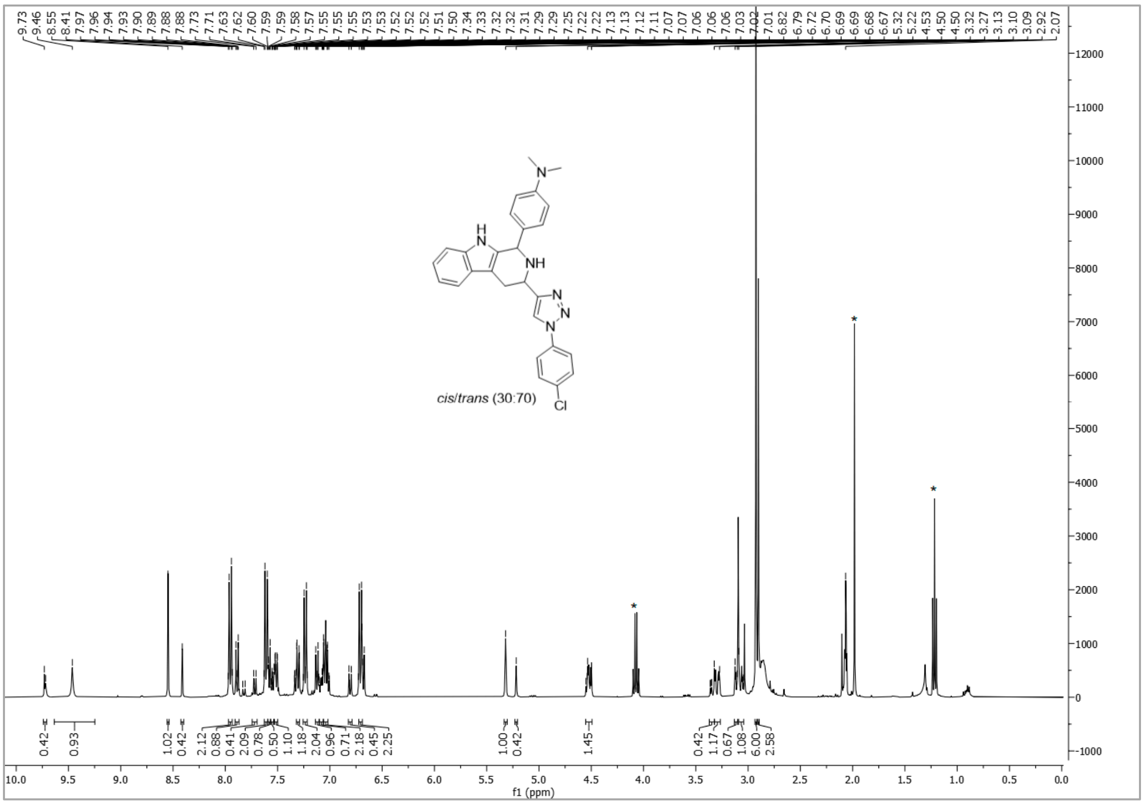

Figure S9. ^{1}H NMR spectrum of compounds **12d** (400 MHz, Acetone-d_6). *Solvent (EtOAc).

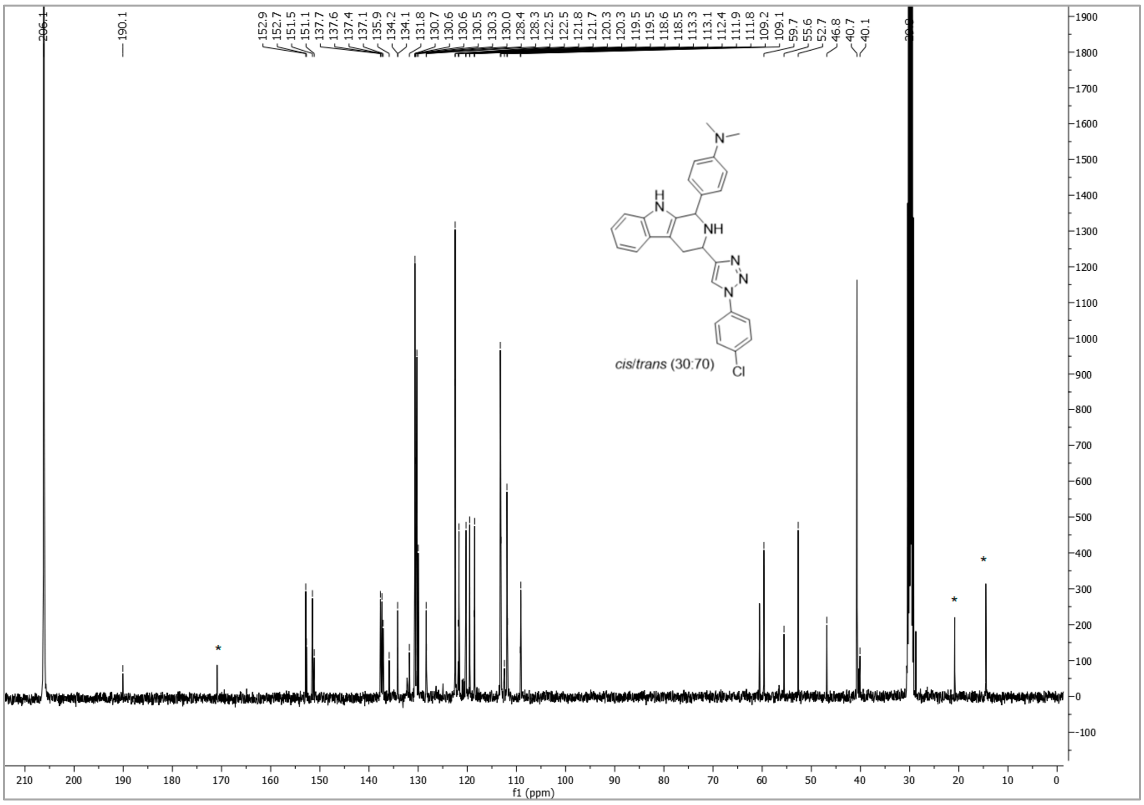

Figure S10. ^{13}C NMR spectrum of compounds **12d** (100 MHz, Acetone-d_6). *Solvent (EtOAc).

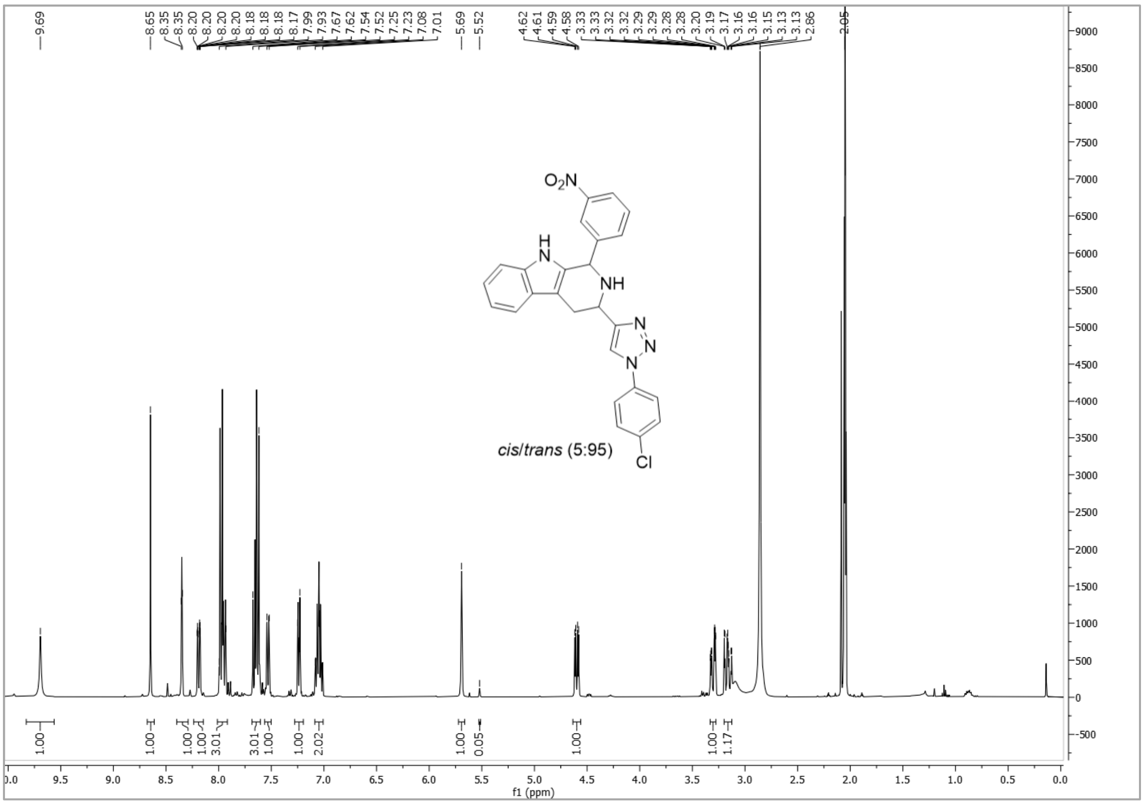

Figure S11. ^{1}H NMR spectrum of compounds **12e** (400 MHz, Acetone-d_6).

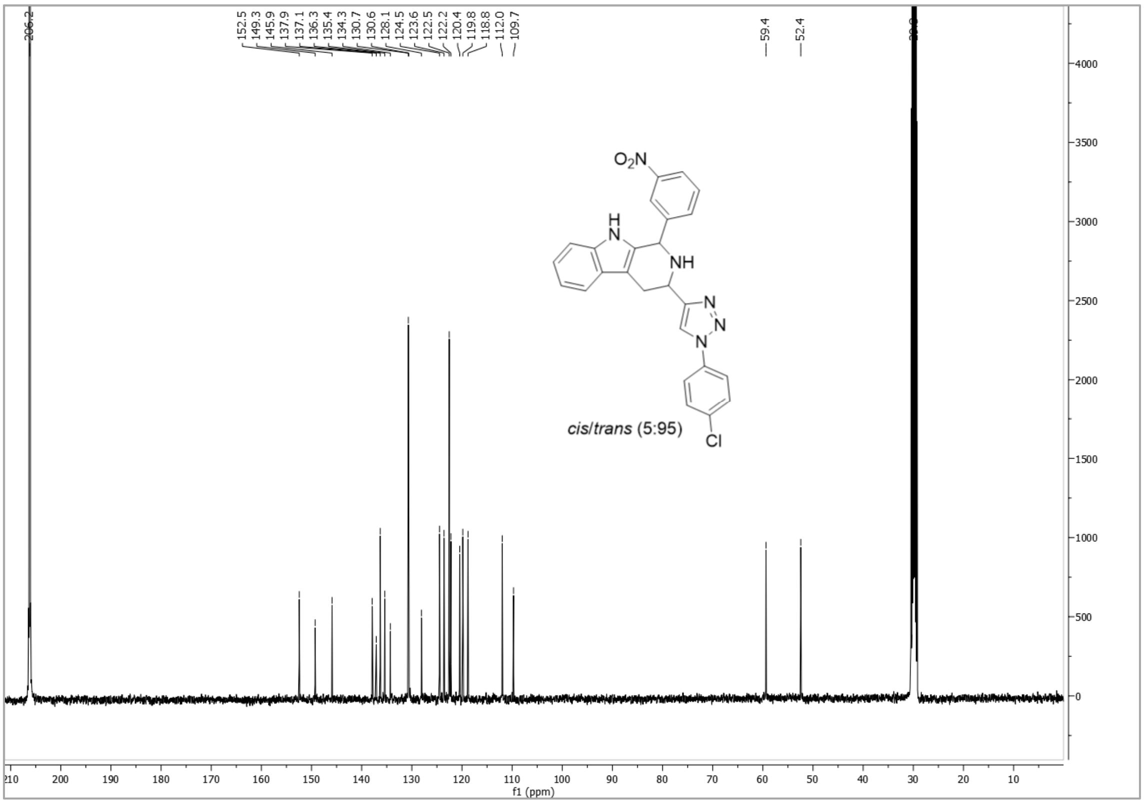

Figure S12. ^{13}C NMR spectrum of compounds **12e** (100 MHz, Acetone-d_6).

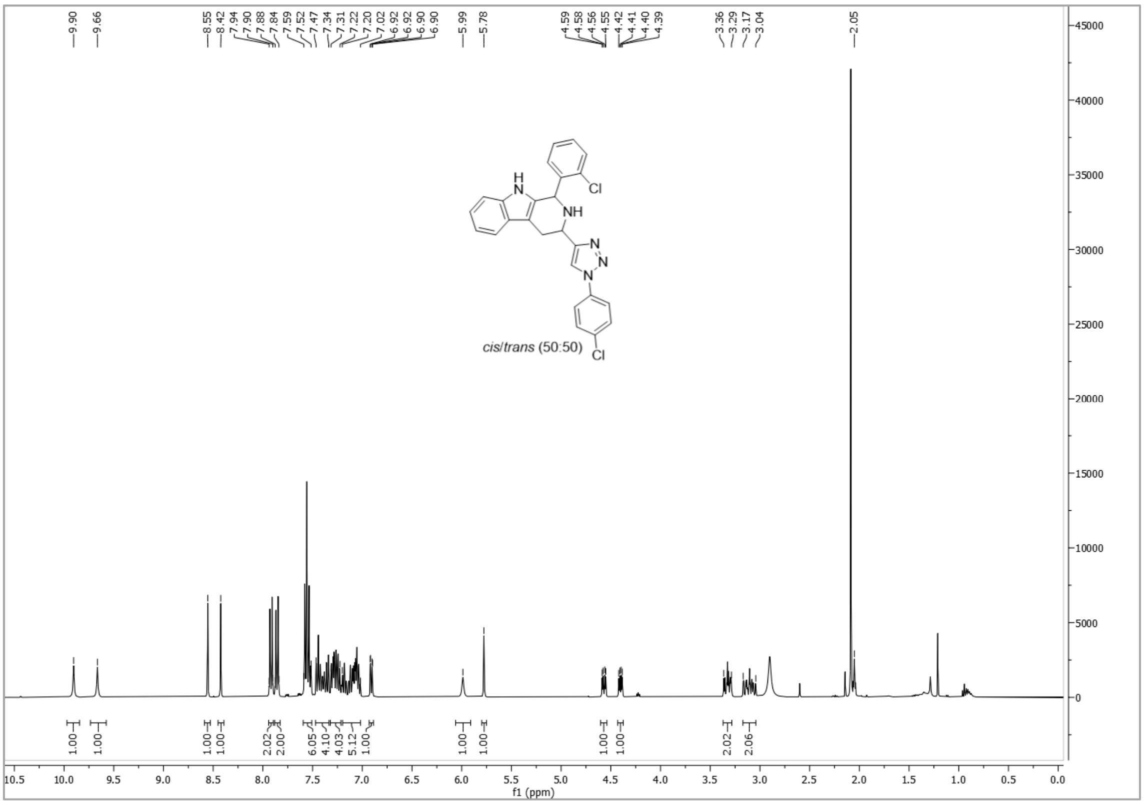

Figure S13. ^{1}H NMR spectrum of compounds **12f** (400 MHz, Acetone-d_6).

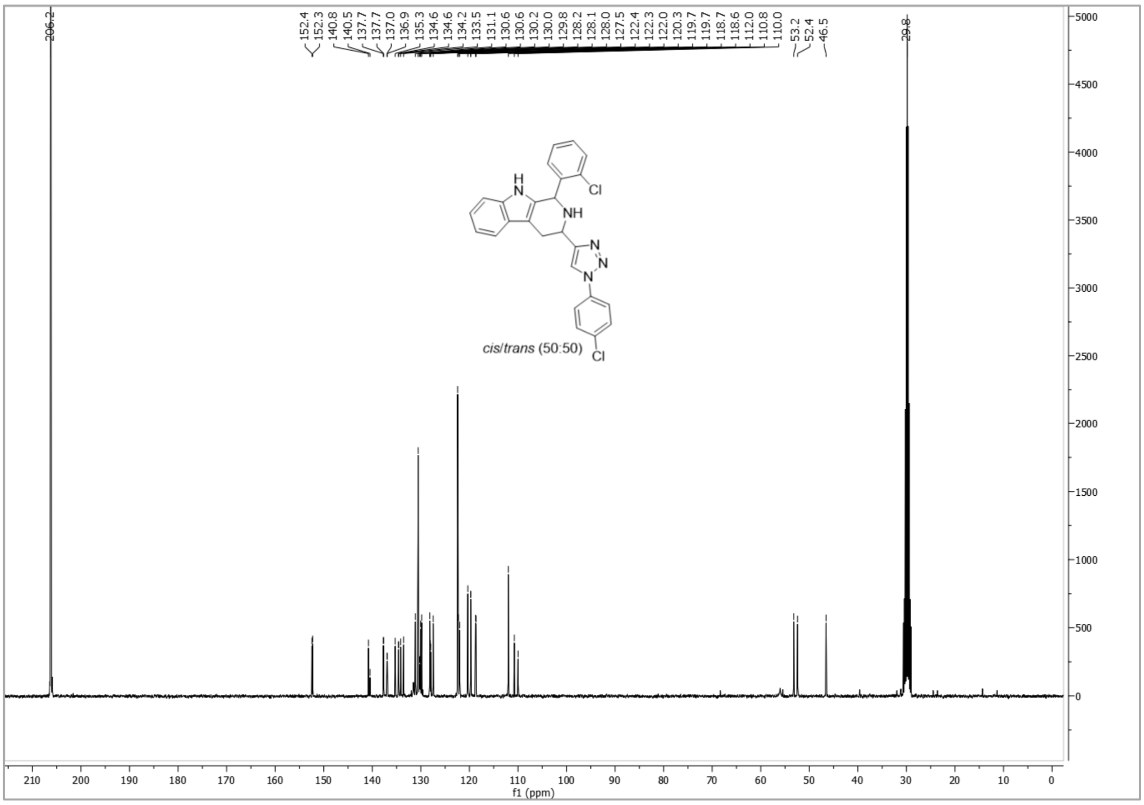

Figure S14. ^{13}C NMR spectrum of compounds **12f** (100 MHz, Acetone-d_6).

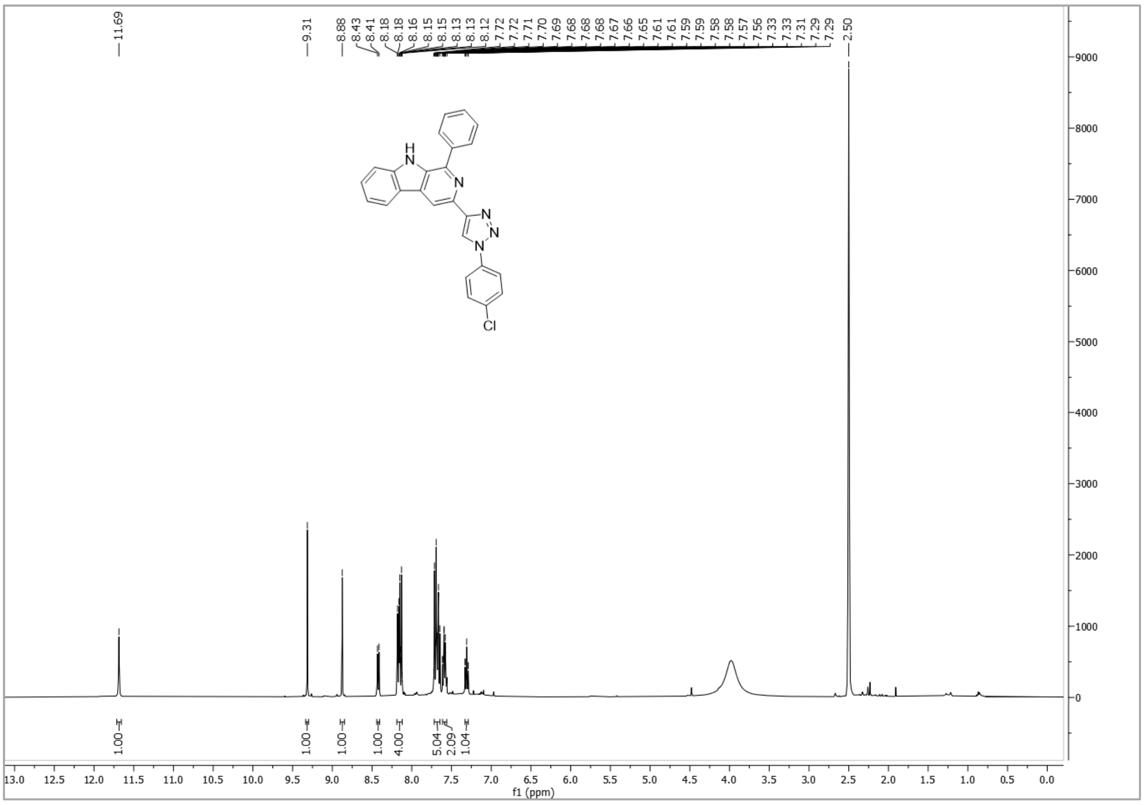

Figure S15. ^{1}H NMR spectrum of compounds **13a** (400 MHz, DMSO-d_6).

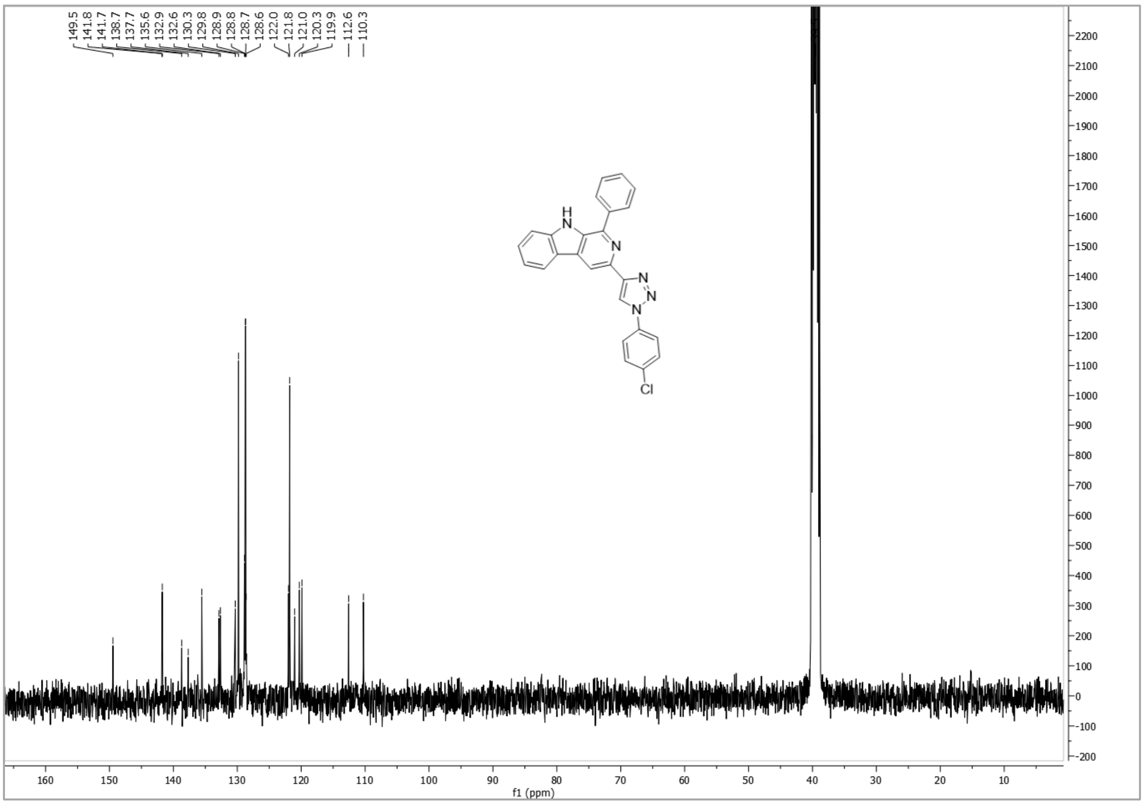

Figure S16. ^{13}C NMR spectrum of compounds **13a** (100 MHz, DMSO-d_6).

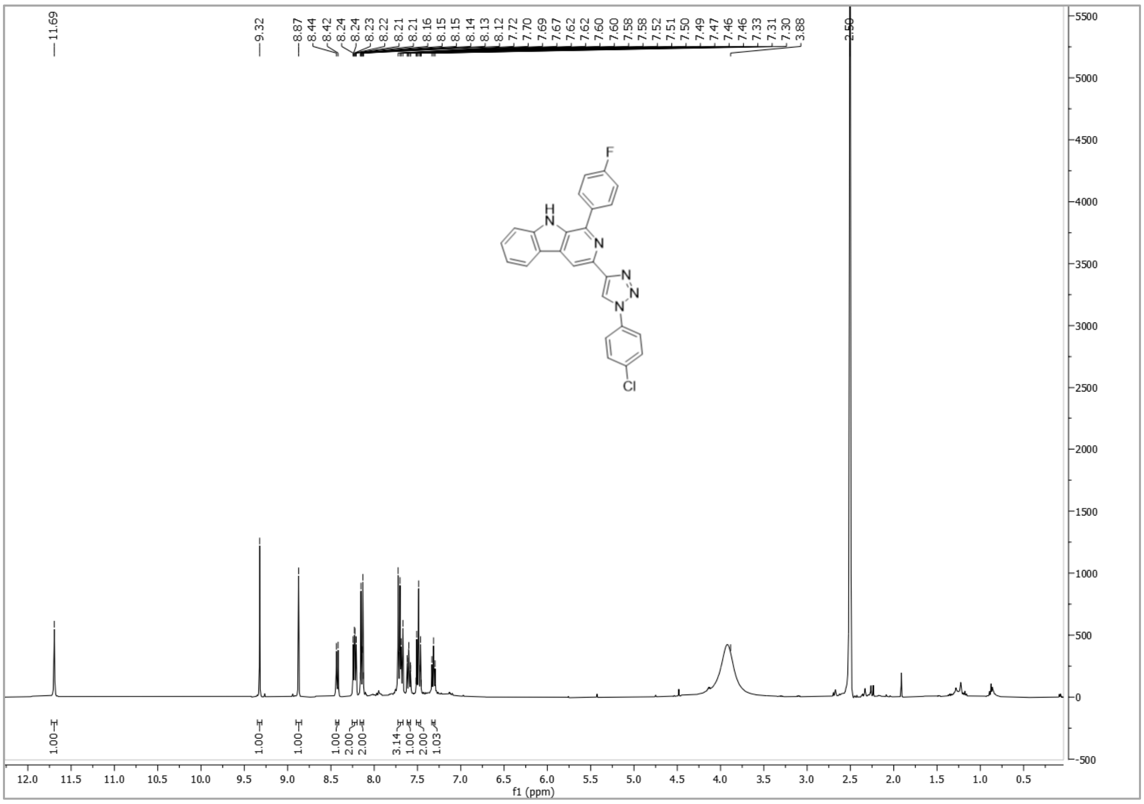

Figure S17. ^{1}H NMR spectrum of compounds **13b** (400 MHz, DMSO-d_6).

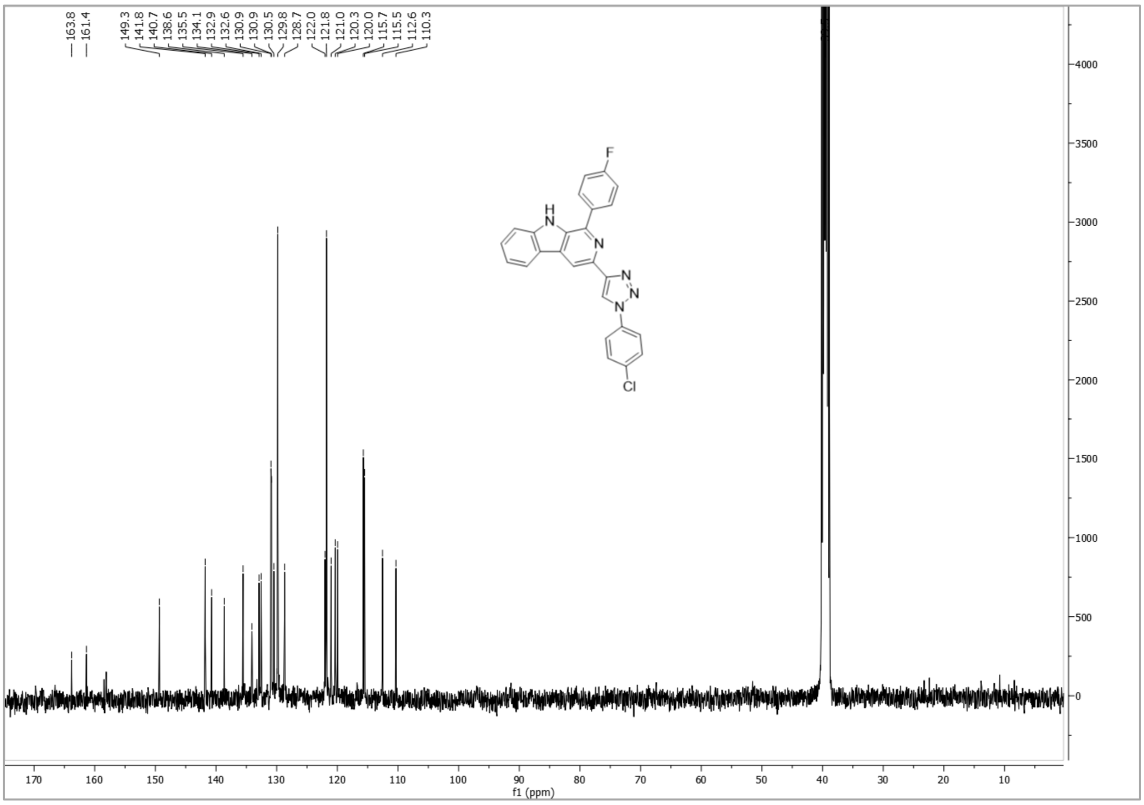

Figure S18. ^{13}C NMR spectrum of compounds **13b** (100 MHz, DMSO-d_6).

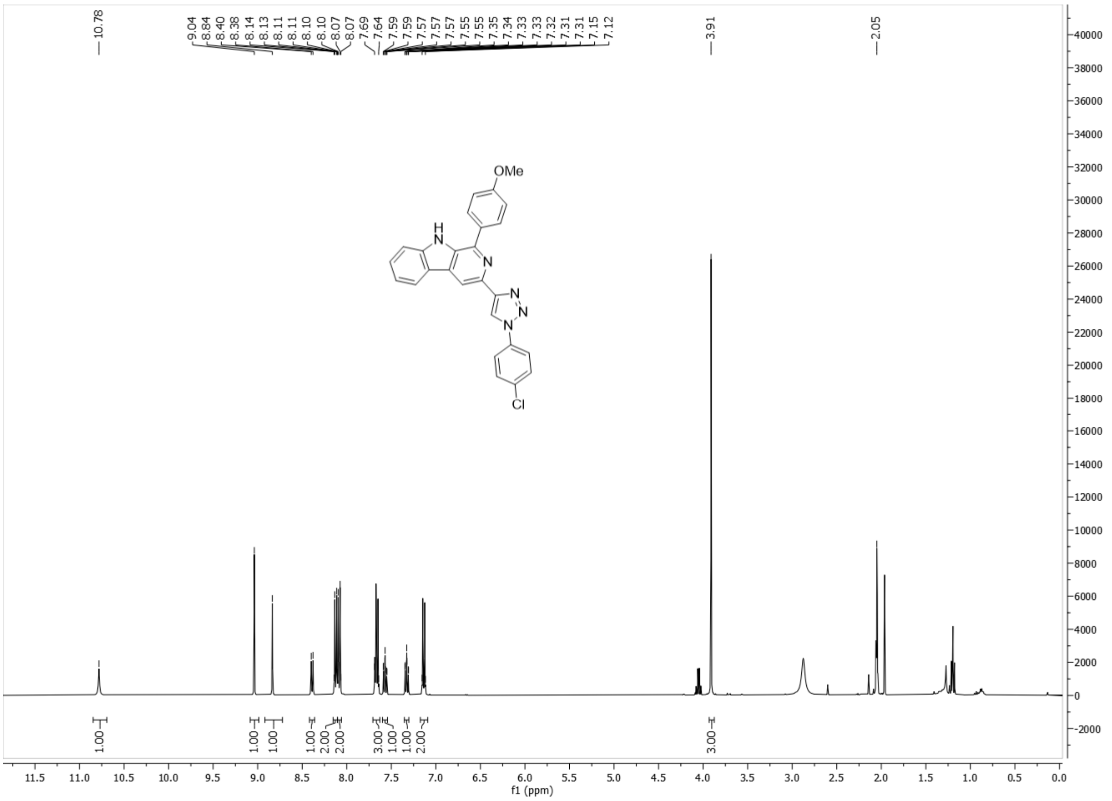

Figure S19. ^{1}H NMR spectrum of compounds **13c** (400 MHz, Acetone-d_6).

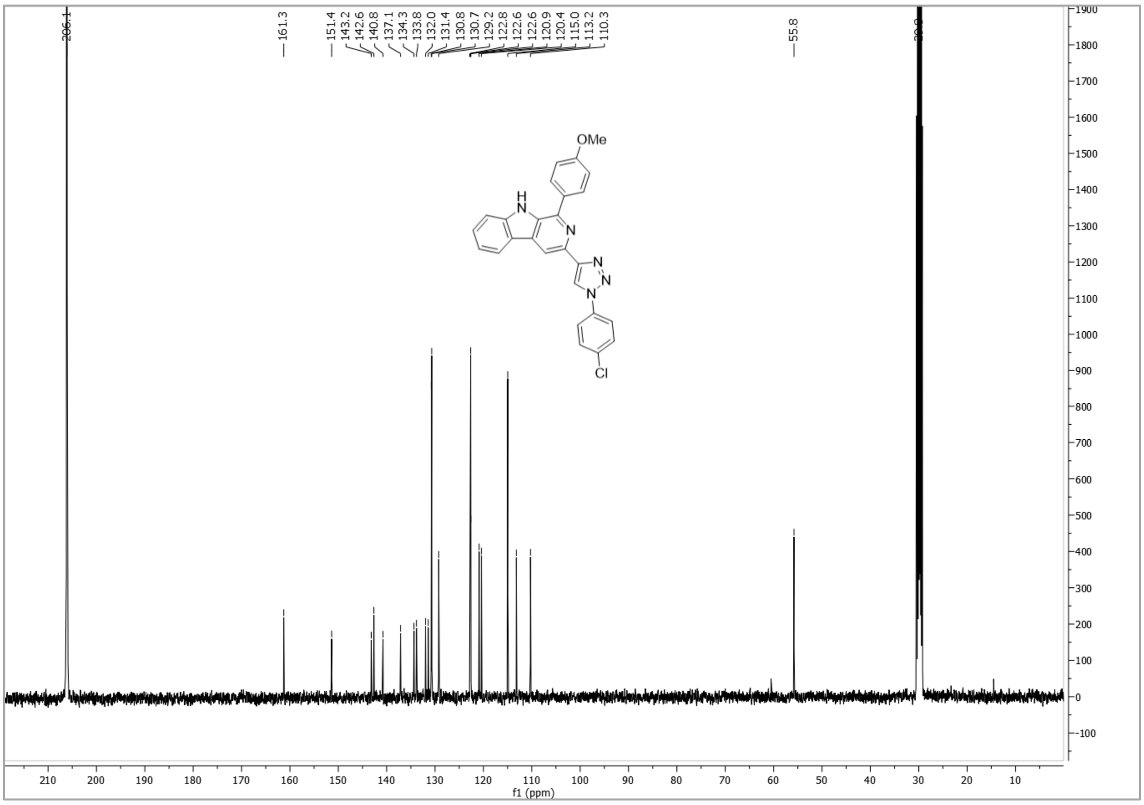

Figure S20. ^{13}C NMR spectrum of compounds **13c** (100 MHz, Acetone-d_6).

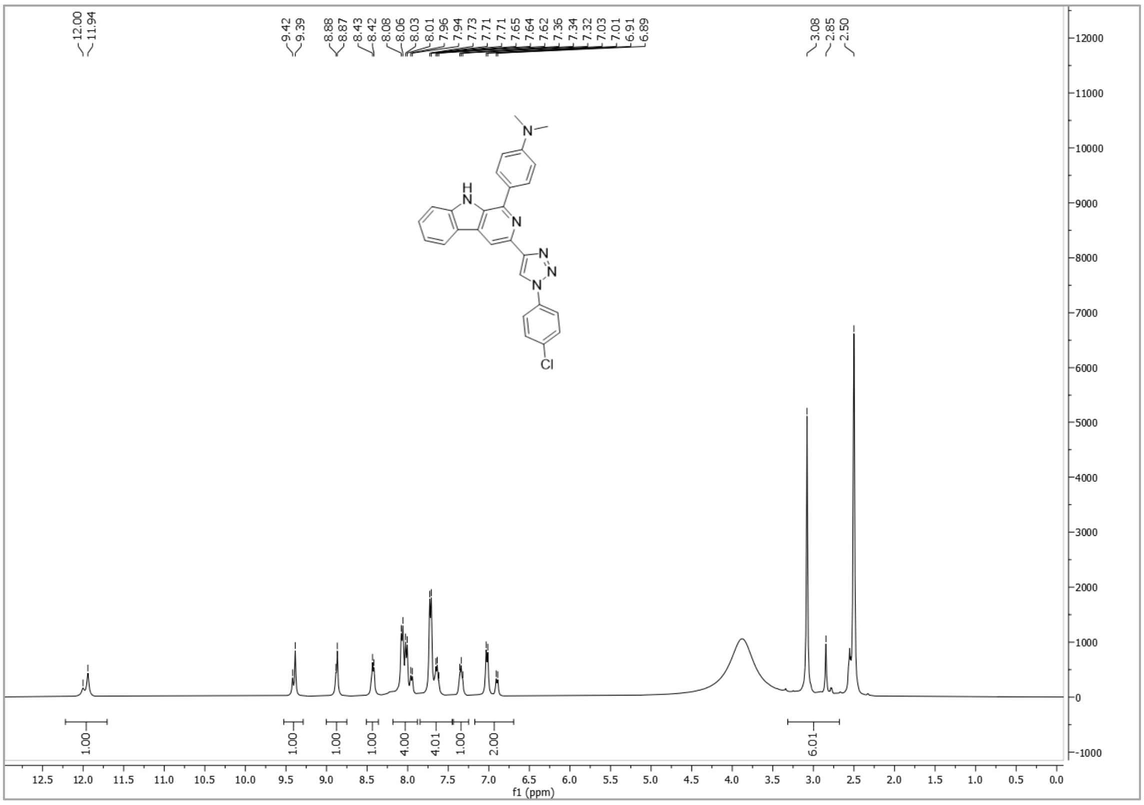

Figure S21. ^{1}H NMR spectrum of compounds **13d** (400 MHz, DMSO-d_6).

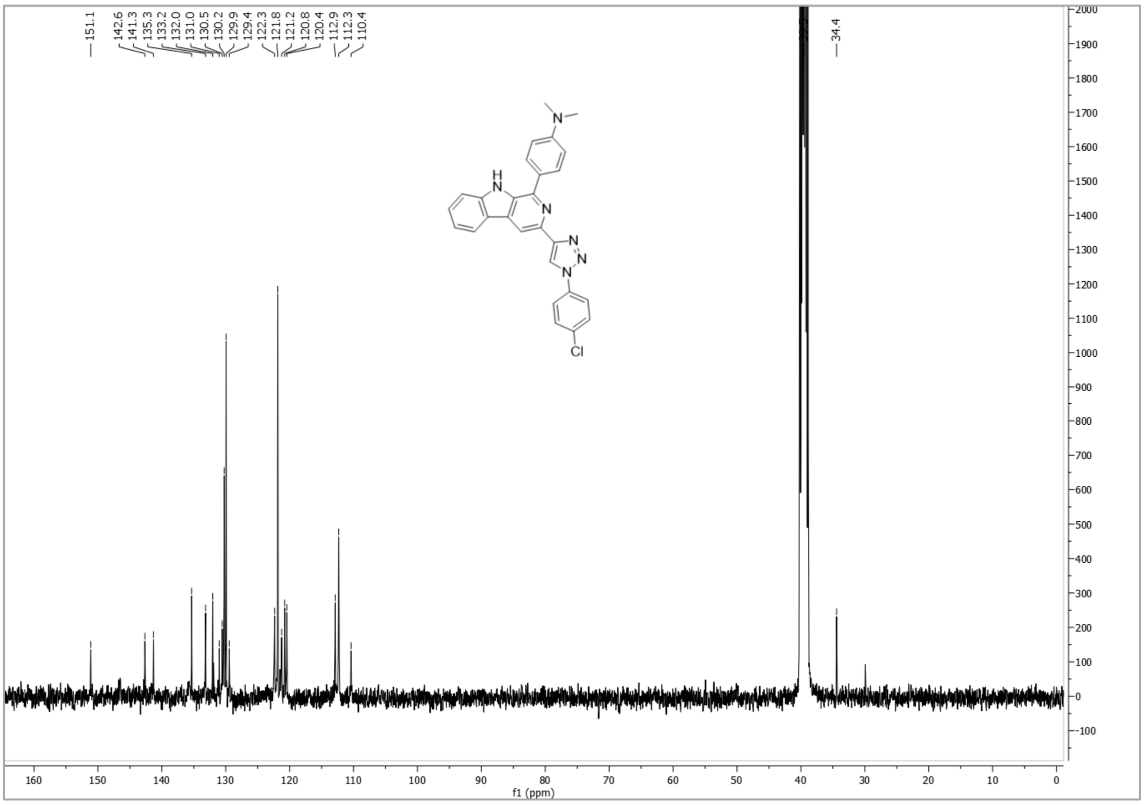

Figure S22. ^{1}H NMR spectrum of compounds **13d** (400 MHz, DMSO-d_6).

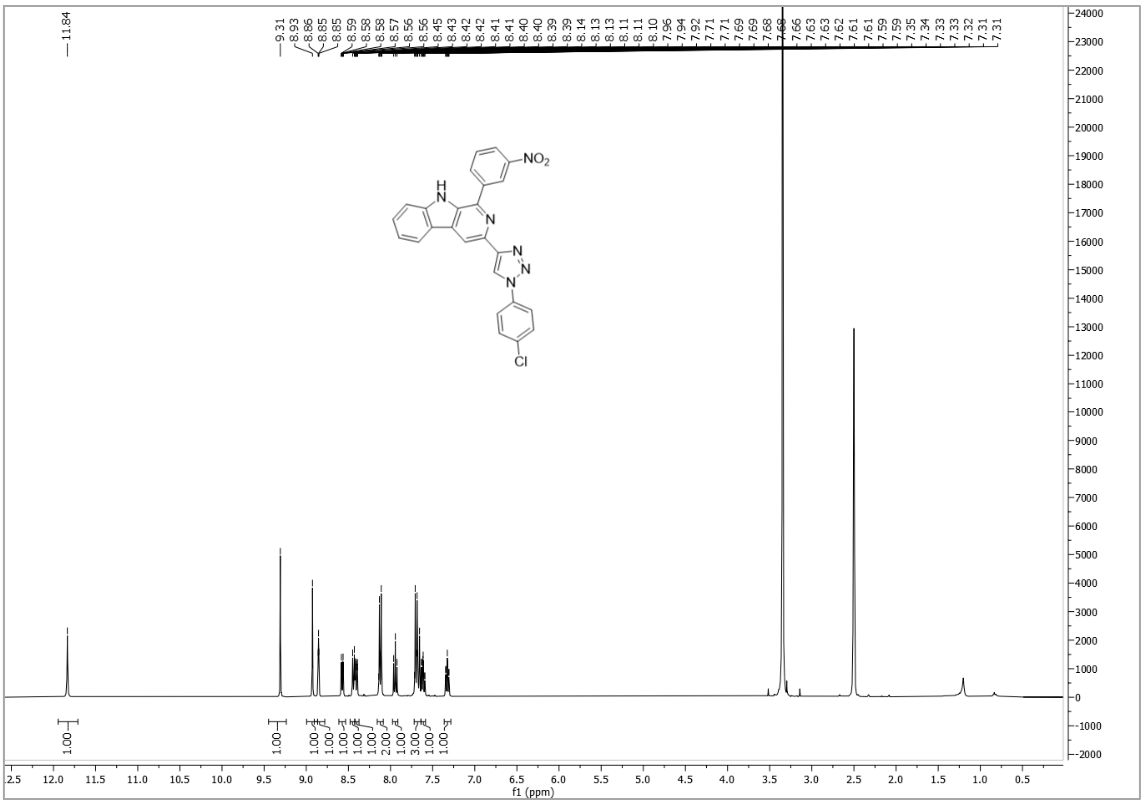

Figure S23. ^{1}H NMR spectrum of compounds **13e** (400 MHz, DMSO-d_6).

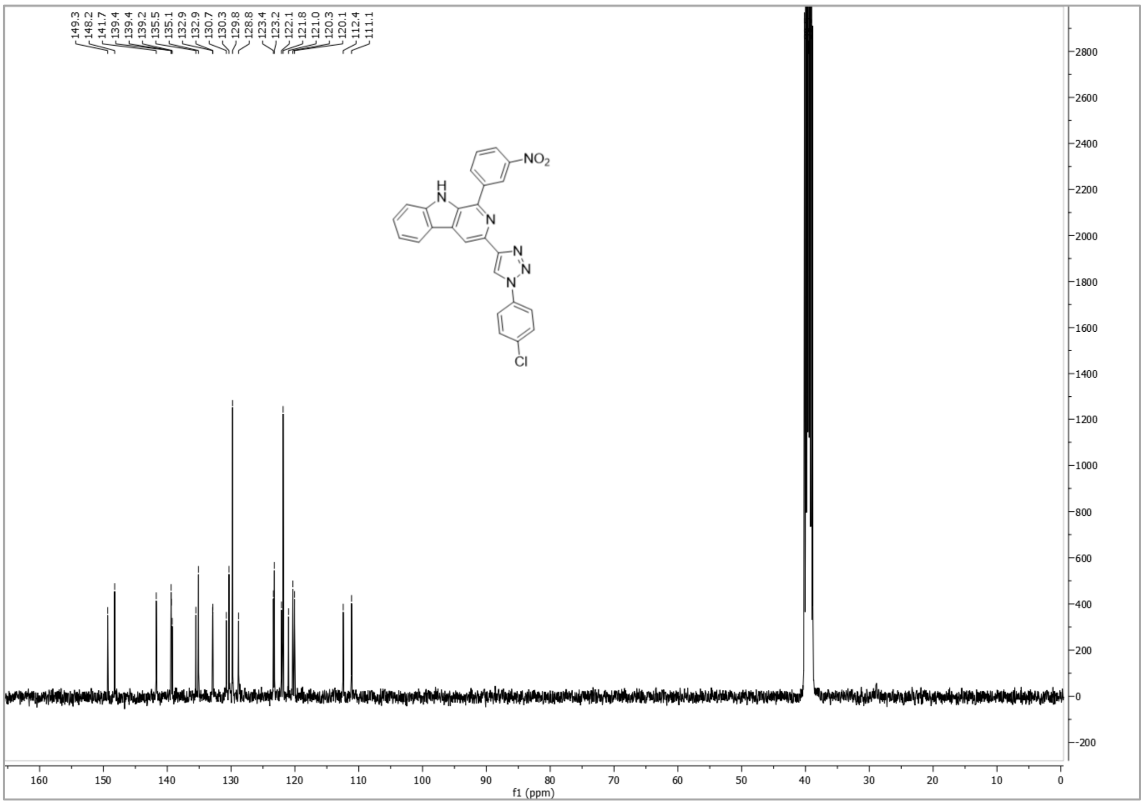

Figure S24. ^{13}C NMR spectrum of compounds **13e** (100 MHz, DMSO-d_6).

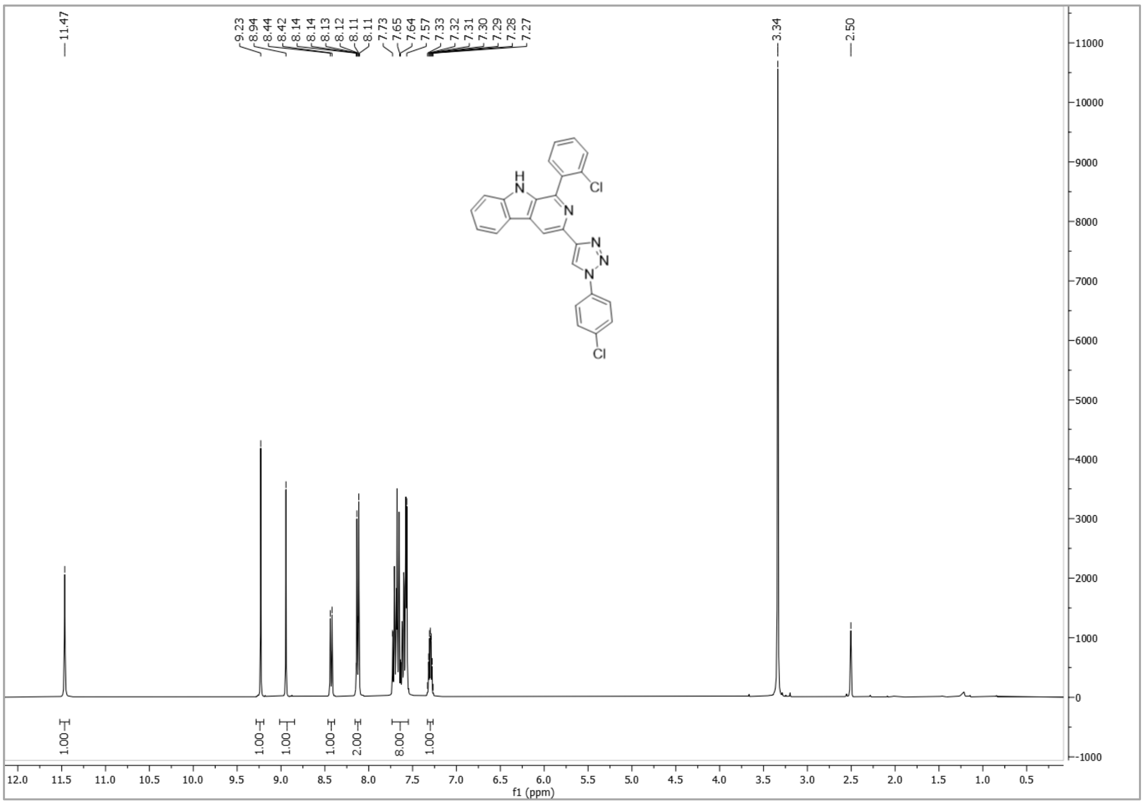

Figure S25. ^{1}H NMR spectrum of compounds **13f** (400 MHz, DMSO-d_6).

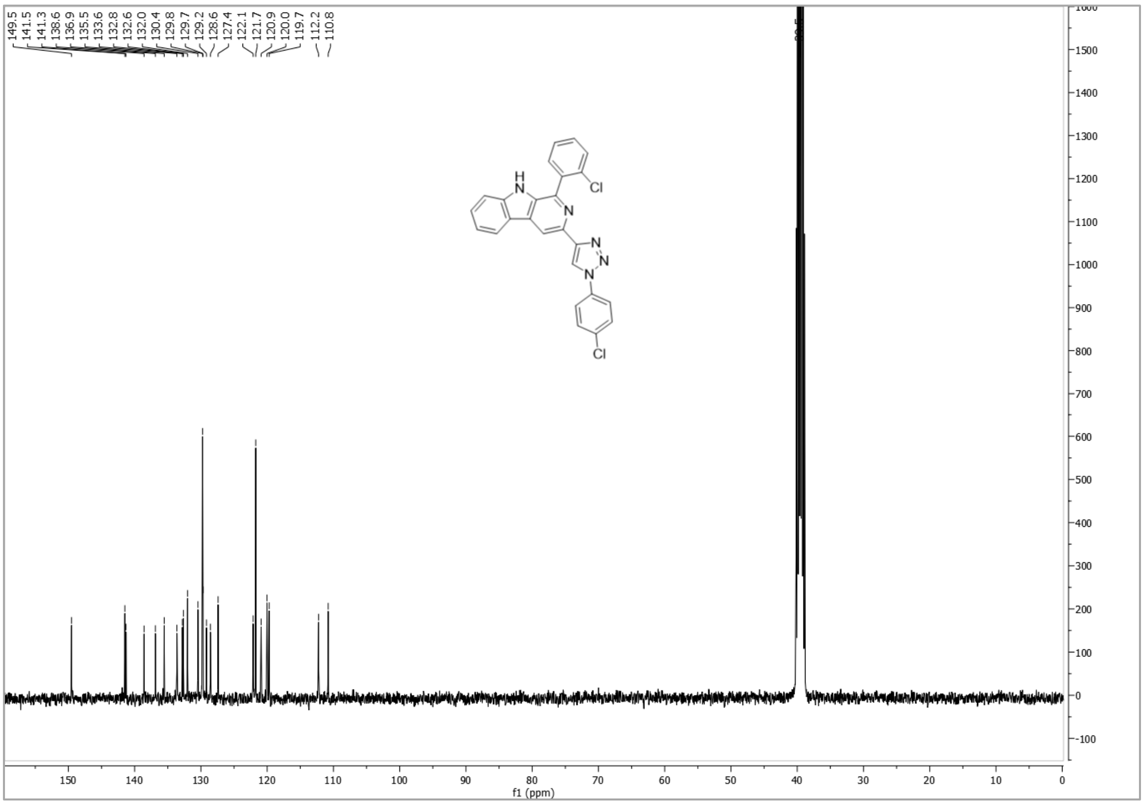

Figure S26. ^{13}C NMR spectrum of compounds **13f** (100 MHz, DMSO-d_6).
